# Scientists' Warning to Humanity: Unnecessary Bureaucracy Is a Global Impediment to Productivity, Advancement of Human and Planetary Wellbeing, Science and Sustainability

**DOI:** 10.1111/1751-7915.70371

**Published:** 2026-05-29

**Authors:** Kenneth Timmis, Zeynep Ceren Karahan, Jennifer A. Byrne, Jake M. Robinson, Patricia Bernal, Paul B. Rainey, Purificación López‐García, Terry J. McGenity, Max Chavarria, Kazuo Sato, Willy Verstraete, Lars M. Blank, Rup Lal, María Francisca Colom, Juan Luis Ramos

**Affiliations:** ^1^ Institute for Microbiology Technical University of Braunschweig Braunschweig Germany; ^2^ Department of Medical Microbiology Ankara University School of Medicine Ankara Turkey; ^3^ School of Medical Sciences, Faculty of Medicine and Health, The University of Sydney, Sydney, & NSW Health Statewide Biobank, NSW Health Pathology Camperdown New South Wales Australia; ^4^ College of Science and Engineering Flinders University Bedford Park South Australia Australia; ^5^ Department of Microbiology Universidad de Sevilla Sevilla Spain; ^6^ Department of Microbial Population Biology Max Planck Institute for Evolutionary Biology Plön Germany; ^7^ Ecologie Systématique Evolution CNRS, Université Paris‐Saclay, AgroParisTech Gif‐sur‐Yvette France; ^8^ School of Life Sciences University of Essex Colchester UK; ^9^ Escuela de Química, CIPRONA Universidad de Costa Rica & Centro Nacional de Innovaciones Biotecnológicas San José Costa Rica; ^10^ Faculty of Education Chiba University Chiba Japan; ^11^ Center for Microbial Ecology and Technology Ghent University Ghent Belgium; ^12^ Institute of Applied Microbiology, Aachener Biology and Biotechnology RWTH Aachen University Aachen Germany; ^13^ Acharya Narendra Dev College University of Delhi New Delhi India; ^14^ Department of Plant Production and Microbiology Miguel Hernández University, San Juan de Alicante, & Institute for Sanitary and Biomedical Research of Alicante Alicante Spain; ^15^ Consejo Superior de Investigaciones Cientificas, Estación Experimental del Zaidín Granada Spain

## Abstract

Bureaucracies are essential to the proper functioning of almost all human activities. However, they are diverse in their quality and success‐promoting potential. While many bureaucratic measures make sense and are necessary for the wellbeing and success of their organisations, some are unnecessary or ill‐conceived/implemented, and yet others may harm system functioning and achievement. Unnecessary bureaucratic measures limit productivity, waste vital resources and drain workforces of engagement, enthusiasm, motivation, commitment and cohesion. (*The term “unnecessary bureaucratic measures” used in this discourse is used in the sense of poor value and lacking a compelling basis*—*cost: benefit; see also GOV.UK 2020*—*but also includes poorly‐* *conceived or ‐implemented measures, measures delegated to frontline and other workers that could/should be handled by the administrations, online tools promoted as reducing workloads but that in fact increase work and frustration because they are poorly designed and/or not subjected to adequate quality control.*) They create frustration, anger and increased stress among those affected. Stress is considered by the World Health Organisation to be one of the foremost health crises of the 21st century. While the impact of unnecessary bureaucratic measures within an enterprise may go unnoticed by those not directly affected, the aggregate negative global impact on productivity, economic success and the wellbeing of workforces is concerning. Humankind is currently faced with a range of challenges, some existential, that urgently require solutions. Scientific research and discovery are generally considered to be major drivers of progress of humankind and a source of solutions to major problems and crises faced by society. Science innovation depends upon the creativity, originality and productivity of scientists. In the fields of biomedical sciences, the plant‐agriculture‐nutrition sciences, and the environmental sciences, progress in the past can be approximately quantified in terms of reduction in preventable human suffering, disease and premature death (as illustrated by the recent development of mRNA vaccines against the SARS‐CoV‐2 virus, which saved millions of lives), as well as the resulting socio‐economic benefits. Unnecessary bureaucratic measures needlessly slow down research progress by diverting the time and effort of scientists from their essential tasks—creative thinking and research—to mundane tasks, and are responsible for delays in humanitarian improvements that translate into avoidable suffering and premature loss of life. Unnecessary bureaucracy is a global crisis of diversion from primary essential tasks, lowered human achievement and ensuing frustration, that is intertwined with and reinforces other problems, such as societal fragmentation. It requires a global response. A new, fit‐for‐purpose framework is needed that supports the functioning of bureaucracies and the necessary measures they impose, while constraining the imposition of unnecessary measures. Here, we consider some of the causes of unnecessary bureaucracy and measures needed to directly address these causes. These considerations lead us to propose an actionable solution strategy involving a Bureaucracy Charter and an implementation mechanism that will ensure best practice and adherence to the Charter. The rationale of this strategy incorporates key issues of benchmarking‐best practice, transparency‐accountability, oversight, stakeholder engagement‐involvement, duty of care, Health in All Policies, and awareness and avoidance of cost externalisations.

## Graphical abstract

Unnecessary bureaucracy is a global impediment to progress and productivity that increases stress and lowers workplace morale and motivation. In research it smothers creativity and innovation that are key to new discoveries and the societal and environmental benefits they catalyse. It is a significant factor preventing attainment of sustainability. Guardrails to prevent unnecessary bureaucracy will consist of robust mechanisms operating in the three stakeholder pillars of individuals, enterprises and bureaucracies, to ensure transparency, accountability, critical scrutiny, benchmarking and oversight of all bureaucratic measures and their implementation.

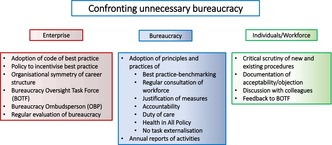

## 1 Introduction

Bureaucracies impose rule‐based order in enterprises, systems and operations and are essential to the proper functioning of most human activities (https://www.britannica.com/topic/bureaucracy). Their purpose is to ensure effective channelling and orchestration of diverse human and other resources towards the achievement of the goals of the enterprise they serve (Nickerson 2024; https://uk.indeed.com/career‐advice/career‐development/what‐is‐bureaucracy). They have a duty to ensure that organisational activities conform to applicable legal, regulatory and ethical standards. Bureaucracies have been essential to productive endeavours since the dawn of civilisation: they existed in ancient Sumer, almost six millennia ago (https://en.wikipedia.org/wiki/Bureaucracy; for a brilliant academic analysis of bureaucracy, see Harari 2024).

So, first things first: *what this Editorial is not*. It is not a critique of bureaucracy per se; it is not a disparagement of administrations, managements and bureaucracies which serve a vital role in the organisation and functioning of enterprises, agencies and civil service and quasi‐civil service. Many bureaucracies function smoothly, are supportive, reasonable and responsive and are a vital asset to the enterprises they serve. Nevertheless, bureaucracies and members thereof collectively exhibit the diversity of quality and success‐promoting potential exhibited by humanity as a whole. Those at the lower end of the scale are perceived by those affected to unjustifiably impose measures lacking obvious value, measures that in some cases may have an overall negative impact on the successful operation of the enterprise or elements of it. Tainter (1988) has proposed that societies eventually collapse under the weight of their own accumulated complexity and bureaucracy. (https://www.bbc.com/future/article/20190218‐are‐we‐on‐the‐road‐to‐civilisation‐collapse), a notion that aligns with Parkinson's Law (in the context of the bureaucratisation of the British Civil Service): “work expands so as to fill the time available for its completion”, and the discourse of Klimek et al. (2008). Although a primary task of bureaucracies is to prevent chaos in the systems they oversee, unnecessary bureaucracy may actually promote it (Abbey 2021).

Bureaucracies have a bad image for a significant fraction of society (e.g., Kohl 2020; Wen 2020), witness the famous phrase “Death, taxes, and red tape. The inevitable trio no one can escape.” (Kaufman 2015; see also Gore 1993; Brewer and Walker 2010), and literature (and indeed the art world: e.g., Nakayama 2002; https://www.dalipaintings.com/the‐average‐bureaucrat.jsp; https://www.wikiart.org/en/george‐tooker/government‐bureau‐1956), is replete with discussions and analyses of bureaucracies (e.g., Tickell 2022), some of which lay out what needs to be improved. However, if solutions are formulated, they mostly focus on relieving symptoms or initiating discussions (e.g., Tickell 2022), rather than exploring the unique contexts of bureaucracies—their ecosystems—that may contribute to problems, and informing how to deal with root causes. Importantly, criticisms and solutions proposed tend to be focused on individual sectors, such as government, education, social care, etc., rather than bureaucracy as a whole, and to ignore the global consequences of bureaucracy's failings.

The purpose of this Editorial is to consider the global impacts of unnecessary bureaucracy and its collateral consequences, and to develop a code of conduct and framework of operation that will promote the supportive roles of bureaucracies and discourage the hindering activities/procedures/strategies that generate much of the irritation and frustration, and that tarnish the image of bureaucracy. In particular, the framework will aim to maximise scientific productivity and its contribution to the solution of global crises and sustainability.

In order to explore potential solutions, we first identify some of the common complaints about bureaucracies, complaints that in extreme cases can reflect dysfunction, counterproductivity and, in rare cases, promote a toxic workplace environment. Evidently, these issues are not characteristic of bureaucracies as a whole, and we do not imply that they are. Moreover, whereas some issues are real, others may simply be perceived—no longer/have never been a problem—and we do not imply that these represent a cause for complaint. However, it is essential to improve the image of bureaucracies and, for this, it is necessary to counter both real and perceived actions, behaviours and attitudes that are the cause of complaint. Secondly, we seek to identify the root causes of the behaviour of bureaucracies that provoke complaints. Thirdly, we develop proposals for actionable remedial policies and processes that address these root causes.

## 2 The Workplace as a Focal Point of Frustration and Dissatisfaction provoked by Unnecessary Bureaucracy

Bureaucracy manages all manner of human activities, but the negative impacts of unnecessary bureaucracy are felt to the greatest extent in the workplace and in interactions with official agencies providing services, such as those related to health and social needs, residency and passports, justice, etc., that are largely run by civil services (Kohl 2020). Public services are usually monopolies and hence cannot be avoided, although exposure to unnecessary bureaucracy meted out by public services is generally occasional and limited in impact. On the other hand, while there is often a choice of workplace that in principle permits escape from uncomfortable environments, most adults spend half of their waking hours at work, so the impact of unnecessary bureaucracy in the workplace can be chronic and cumulative.

A workplace usually has a primary purpose, namely, to achieve something that contributes to the success of the enterprise/organisation. This may be to produce cars, deliver mail, teach children, advise on investments, cure diseases, prevent pollution of the environment, prevent public exposure to contaminated food, etc. Organisations have people directly involved in creating and delivering (including promoting and selling) the product or service it provides—its primary producers or frontline staff—and support staff, administrations and management. The purpose of the support staff, administrations and management is generally to optimise the output and efficiency of the enterprise—qualitatively and quantitatively—to achieve its success, frequently through optimising the efficiency and productivity of frontline staff (Spector 2019).

### 2.1 Unnecessary Bureaucracy Reduces the Time and Effort Spent on Primary Tasks and Compromises the Success Potential of Organisations

While there are many indicators of the success of an enterprise, an important one is the relationship between resources consumed, principally money, and production output and its value. In many enterprises, the major cost is salaries. How effectively employees use their time is a measure of how effectively an enterprise uses much of its money. While in principle all members of an organisation contribute to its success, a rate‐limiting parameter in productivity is often time spent by the front‐line individuals, who create and/or deliver the product of the enterprise (e.g., in the case of a research enterprise, the researchers and their technical staff), on their primary task/role (Allgood and Perez 2025). Spending time on *unnecessary* bureaucracy‐administration can *unnecessarily* divert effort away from production‐delivery and hence is a waste of personnel and the financial resources they represent (e.g., Spector 2019), reduces productivity‐delivery and degrades the success potential of the enterprise. Since many organisations operate in a competitive environment, research organisations included, and despite the high degree of collaboration that characterises research, those permitting unnecessary bureaucracy unnecessarily inflict self‐harm by creating competitive disadvantage (See Murthy 2022).

### 2.2 Unnecessary Bureaucracy Is a Cause of Workplace Frustration and Dissatisfaction

Many, if not most, fresh new employees start with considerable enthusiasm, creativity, commitment and high expectations, in part due to youth and created by the new environment and the excitement of a new learning curve (see, e.g., Slim 2020). The challenge is to nurture this enthusiasm—belief in and ownership of the tasks conferred on the individual—which is germane to the productivity, team spirit, health and success of the enterprise, against a backdrop of falling novelty and the normal frictions and disappointments that may occur over time in the workplace and personal life. An increasing workload of bureaucratic tasks whose necessity is neither obvious nor plausibly explained, that take up an increasing amount of time, and that divert effort from the primary tasks and diminish the personal satisfaction experienced through achievement, can lead to frustration (e.g., see Faragher et al. 2005; Scott et al. 2021) and drain employees of enthusiasm, engagement and commitment. A lowering of engagement can result in reduced productivity, over and above that lost through time spent on bureaucratic tasks, and lower morale and team spirit, sour relationships and reduce social cohesion in the workplace, and the health and coherence of an enterprise (Gallup 2025).

### 2.3 Workplace‐Induced Frustration and Dissatisfaction Can Cause Stress

Frustration can have diverse causes, including unnecessary bureaucracy, poorly conceived, badly communicated or unnecessary procedures, as well as the difficulty associated with navigating across participating entities whose processes are not joined up, and for which no one takes overall responsibility. The greater the intensity of frustration and dissatisfaction, the more likely they will be manifested in stress. Chronic frustration may lead to chronic stress. Stress is exacerbated when there is no clear means to report and fix such issues. Unnecessary bureaucracy‐engendered loss of time to complete primary tasks may also lead to a build‐up of unfinished work, which may compound the stress.

While some individuals are able to mentally compartmentalise their work and private lives and avoid spillover of frustration and stress between one and the other, many cannot and problems in one sphere affect the other. The extended time spent in the workplace constitutes extended exposure to and heightened impacts of its influences, in particular satisfaction, but also frustration and dissatisfaction: the consequences of psychosocial factors (International Labour Organisation 2016; Leka and Jain 2024). Stress is experienced differently among individuals according to personal susceptibilities and the degree of frustration and dissatisfaction caused or perceived.

### 2.4 Workplace Stress Is a Significant Cause of Health Disorders

Workplace stress is the second most frequent workplace health issue (Milczarek et al. 2009; WHO 2003, 2024; Maulik 2017; Leka and Jain 2024). It has been estimated that workplace stress causes 120,000 deaths in the US each year (Goh et al. 2015), so the global death rate will be substantial (Murthy 2022). Given that stress‐related morbidity and its collateral impacts on the wellbeing of family and friends will be significantly higher than mortality, the global health impact of workplace stress will be huge. The OSH Pulse Survey published by EU‐OSHA in 2025 suggests that almost 30% of workers experience stress, anxiety or depression caused or made worse by work (https://osha.europa.eu/sites/default/files/documents/OSH‐pulse‐2025‐mental‐health_infographic_EN.pdf).

Excessive and prolonged stress can engender a number of disorders (Selye 1956, 1976; https://www.osha.gov/sites/default/files/Long‐Term_Stress_Harms_Workplace_Stress_Toolkit_revised_508.pdf; de Kloet et al. 2005; Meineke and Gordichuk 2024; Hornstein and de Guerre 2006; McEwan 2008; Yaribeygi et al. 2017; International Labour Organisation 2016; https://osha.europa.eu/en/themes/psychosocial‐risks‐and‐mental‐health; Mbiydzenyuy et al. 2024; Zafar et al. 2021), including

- Physical health impairments, such as cardiovascular, metabolic, gastrointestinal and musculoskeletal disorders (e.g., Liu et al. 2017).
- Sleeping problems (e.g., Kalmbach et al. 2018) and eating disorders.
- Mental health challenges, such as anxiety, depression, heightened emotions‐anger‐aggression, sexual dysfunction, reduced concentration and motivation, difficulty with memory and problem‐solving, social withdrawal (e.g., Kruk et al. 2004; de Kloet et al. 2005; Craig 2007; Haller 2022).
- Coping behaviours, such as alcohol and drug abuse, smoking, unhealthy diet, insufficient physical activity, sleep disorders: allosteric overload (e.g., Taylor and Stanton 2007; McEwan 2008; Reinke et al. 2025).
- Suboptimal workplace behaviour, including increased absenteeism, decreased ability to recognise and avoid accidents, reduced productivity, lower morale and increased tendency to change employer (https://www.osha.gov/sites/default/files/long‐term_stress_harms_workplace_stress_toolkit_revised_508.pdf).
- Collateral effects of stress‐related disorders, such as decreased family cohesion that can, for example, cause behavioural and emotional problems, academic decline in offspring, caregiver fatigue, dysfunctional relationships in social networks and erosion of social support, stigmatisation, poor management of family finances resulting in increased economic burdens (Greenhaus and Beutell 1985; Westman 2002; Frone 2003; Sandi and Haller 2015; Repetti and Wang 2017),
- Human suffering, something that is often neglected but experienced as a serious deterioration of quality of life by those directly affected and the immediate members of their networks (Maslach et al. 2001).

### 2.5 Workplace Stress Is a Significant Cause of Loss of Productivity

The World Health Organisation (WHO) estimates that 12 billion working days are lost every year to depression and anxiety at a cost of US$ 1 trillion per year in lost productivity (WHO 2024). In 2002, the annual economic cost of work‐related stress in the EU15 was estimated at EUR 20 billion (European Commission 2002).

It should be noted that bureaucrats also experience varying degrees of stress which, in general, also correlate with poorer performance, thereby exacerbating any negative interactions with the workforce they serve, and reducing bureaucratic value itself (Mikkelsen et al. 2024).

**Constructive and destructive stress: eustress and distress**

Many forms of employment impose some degree of stress, not all of which is unhealthy. There is constructive stress, often associated with collective workforce effort and achievement, which may improve performance and provide satisfaction, and may increase system cohesion and ownership: *eustress* (Selye 1974). But there is also destructive stress that entrains frustration, reduces satisfaction, reduces system cohesion, and is fundamentally unhealthy: *distress* (Selye 1974). Unnecessary bureaucracy that reduces productivity generally results in *distress*.

### 2.6 Is Unnecessary Bureaucracy a Global Cause of Ill Health?

Since stress is known to be a predisposing factor for a range of neuropsychiatric‐psychosocial and physical disorders, it would not seem to be unreasonable to assume a link between the stress promoted by unnecessary bureaucracy and ill health. This certainly seems to be the case for professions characterised by high stress. One of these is farming which “ranks in the top ten job groups with a high mortality from suicide.” According to one report “Whilst many farmers cited financial problems as a major cause of stress, changing regulations and paperwork remained the most important stressor identified. The burden of paperwork was identified as a key stressor from all strands, either by understanding or completing the forms or the amount of paperwork involved. These results concur with those of the NFU who stated that several actions still needed to be taken to further reduce the burden of bureaucracy.” (Walsh et al. 2016). Another high‐stress occupation is mental health work with military personnel suffering from post‐traumatic stress in the US Veterans Administration, work that may be associated with “compassion fatigue” and frequently leads to burnout (Maslach and Leiter 2016) among treating clinicians. However, a study of burnout in these clinicians revealed that its main cause was not “compassion fatigue” but rather “perceptions of organisational politics and bureaucracy, as well as their overall workload” (Garcia et al. 2014; https://www.research.va.gov/currents/spring2015/spring2015‐4.cfm; see also Faragher et al. 2005).

Individuals in occupations not considered to carry exceptional levels of stress can also suffer from bureaucracy‐linked ill health (Hornstein and de Guerre 2006) and issues such as burnout, elevated stress, emotional exhaustion and psychiatric symptoms are linked to bureaucracy in the literature (e.g., see Abdelhay 2024; Hornstein and de Guerre 2006; Herd and Moynihan 2021; and references therein). The WHO (2024) specifies that “Risks to mental health at work can include:” “under‐use of skills”, “lack of control over job design or workload”, and “unclear job role”. Similarly, the UK NHS specifies “a lack of control of our workload”, and “high demands on our time and energy and lack of clarity about responsibilities” as two of the seven main causes of stress at work (https://www.nhs.uk/every‐mind‐matters/lifes‐challenges/work‐related‐stress/). According to large‐scale analyses carried out by the Gallup Organisation (2025), high employee engagement is reflected in 70% higher wellbeing. Thus, wellbeing is linked with engagement which suggests that parameters that reduce employee engagement, such as frustration caused by unnecessary bureaucracy, reduce wellbeing (see also Tennant 2001; Gordon and Schnall 2009). Given the global scale of unnecessary bureaucracy, it is vital that causalities be clarified and quantified, in particular the potential link between unnecessary bureaucracy, stress and stress‐induced health disorders.

### 2.7 Can Unnecessary Bureaucracy Constitute “Structural Violence”?

Structural violence is *avoidable* harm caused by social structures or institutions that prevent people from meeting their basic needs or rights (Galtung 1993). Although there is considerable discussion about what constitutes structural violence and basic needs/rights, common elements include systemic/structural contexts in which some people live that unnecessarily prejudice their health, personal development and dignity. It often reflects asymmetry of power between groupings within society, exploitation of such power, and discrimination. Bureaucracy is a structural/systemic component of daily life that embodies asymmetry of power and that often preferentially negatively affects certain subgroups of society, namely frontline workers (as opposed to management and bureaucracies themselves), the infirm and others.

Human dignity is a fundamental right recognised in the United Nations Universal Declaration of Human Rights (https://www.un.org/en/about‐us/universal‐declaration‐of‐human‐rights) and the European Union Charter of Human Rights (https://fra.europa.eu/en/eu‐charter/article/1‐human‐dignity). Excessive bureaucracy and managerialism reduce dignity by unnecessarily restricting the autonomy of those affected and fostering impersonal behaviour in bureaucracies (Klikauer 2013). Weber developed the “iron cage” and “mechanised petrification” metaphors to convey the image of dehumanisation, loss of freedom of thought, and the stifling of creativity and innovation (https://plato.stanford.edu/entries/weber/#IronCageValuFrag; Adler and Borys 1996; DiMaggio and Powell 2000; Ryan and Deci 2000). Even essential bureaucracy, when practiced sub‐optimally or unprofessionally to enjoy the use of power, to create rather than to solve problems, can impact human dignity. As we discuss below, old, infirm and other disadvantaged members of society often have difficulty with bureaucratic procedures, difficulties that may induce/amplify frustration and stress, reduce the ability to achieve what they need or seek, and also reduce their dignity. They may be subjected to unwarranted criticism for their difficulty in completing bureauctratic tasks readily accomplished by younger, healthier members of society. Importantly, this constitutes a pervasive form of discrimination that differentially affects diverse societal groups (Kohl 2020). Even among the fit and able, frustration, dissatisfaction and stress associated with unnecessary bureaucracy can constitute an degradation of wellbeing in those affected. Unnecessary bureaucracy might thus be considered to constitute structural violence.

## 3 Unnecessary Bureaucracy Having Extended Impacts

While the direct negative impacts of unnecessary bureaucracy in an organisation are mostly experienced locally, within an organisation and by immediate family‐friend networks of those affected, the effects of certain types of organisation‐profession may have considerably greater, in some cases global, reach. Examples include *inter alia* education, healthcare, and research organisations.

### 3.1 “Teaching Is the Profession That Creates All Others” (Unknown)

Education is often superficially considered to be primarily the communication of knowledge and understanding relevant to the future needs, health, enjoyment and success of the lives of learners and society. However, its greater purpose in many cultures is to develop the minds and nurture the abilities of learners—to enable, support and encourage them to fulfil their full potential, to stimulate curiosity and a desire for discovery, to promote independence of thought and development of critical and systems thinking and cognitive skills, and promote social and societal engagement, dialogue and problem‐solving with others. This generally includes character building: development of moral and ethical values and a sense of collective responsibility/citizenship, cooperation, social justice and sustainability. Inherent in most concepts of education is the betterment of humanity (https://www.ohchr.org/en/resources/educators/human‐rights‐education‐training/12‐integrated‐framework‐action‐education‐peace‐human‐rights‐and‐democracy‐1995; https://www.unesco.de/assets/dokumente/Bildung/01_Bildung_allgemein/Peace_Education_in_the_21st_Century.pdf; Timmis et al. 2024). Education is the foundation of many human endeavours and its quality ultimately determines the quality of outcomes of such endeavours. Since many children spend more of their waking hours at school than with their parents, teachers can play a major role in the development of their moral‐ethical values. Some of the capabilities and quality of policies and actions of leading members of society, like politicians, captains of industry, etc., and the influence these have on others, may reflect the quality of teaching they received in school.

Excessive bureaucracy in education reduces educator time available for primary tasks, including lesson preparation, reflection on individual student and class needs and consideration of activities to address these needs (see Husain 2024). It reduces teaching quality and opportunities to discover and nurture inherent talents. Teaching, like healthcare and research, is considered by some to be more of a vocation than a profession, which may account for the fact that teachers work more overtime without pay than employees in any other type of work (TUC 2025; https://www.theguardian.com/education/2024/feb/23/daylight‐robbery‐two‐in‐five‐uk‐teachers‐work‐26‐hours‐for‐free‐each‐week). This, coupled inter alia with “poor management practices” (Mehta 2013; Arnold and Rahimi 2025), causes dissatisfaction, frustration and stress, in some cases burnout, which results in a high rate of loss of teachers (Reinke et al. 2025; https://www.theguardian.com/education/2024/feb/26/bureaucracy‐is‐ruining‐teachers‐lives). Teacher turnover in turn disrupts student learning, reinforcing quality deterioration in education due to other causes (https://www.winginstitute.org/teacher‐retention‐turnover#:~:text=Teacher%20turnover%20is%20also%20detrimental,vacated%20by%20less%20effective%20teachers).

According to one report, there are around 94 million teachers worldwide (https://teachertaskforce.org/what‐we‐do/Knowledge‐production‐and‐dissemination/data‐teachers; https://databrowser.uis.unesco.org), with another 44 million needed by 2030 (https://teachertaskforce.org/sites/default/files/2024‐10/1682‐24_FactsheetTeachers_WEB.pdf). The global importance of teachers for all spheres of human activity is immense; the global impact of unnecessary bureaucracy on education is equally immense.

### 3.2 Healing Reduction in Healthcare

According to Boniol et al. (2022), the global health and care workforce in 2020 exceeded 65 million workers and is projected to rise to 84 million by 2030 (WHO 2025a, 2025b). Another source suggests that the health and social care sector accounts for around 13% of total global employment (https://zipdo.co/job‐industry‐statistics/). In economic terms, one estimate of the value of the healthcare market in 2025 was in excess of US$ 13,000 billion (https://www.globalgrowthinsights.com/market‐reports/healthcare‐market‐112708), and another estimated it to represent about 10% of global GDP (https://gitnux.org/global‐healthcare‐industry‐statistics/).

As in other organisations, unnecessary bureaucracy takes up valuable time of frontline health professionals, which reduces their availability for timely diagnosis and treatment of patient disorders and can lead to poorer health outcomes (see also Timmis and Timmis 2017). This was stated unambiguously by the UK Department of Health and Social Care in its “Busting bureaucracy” paper (GOV.UK 2020): “…*excess bureaucracy reduces the time that staff have for care and hinders staff and leaders from deciding how to manage risk, being creative, innovating to fix problems, empowering others and being flexible. This negatively impacts staff well‐being, morale and retention while hindering the very outcomes the processes aim to support*.” (see also Munro 2011). According to Herd and Moynihan (2021; see also references therein), *one study found that physicians spent twice as much time on paperwork as they did with patients. Dealing with these administrative hassles is a leading cause of physician burnout* (https://www.ncbi.nlm.nih.gov/books/NBK552615/). This is exacerbated in some healthcare systems by metric‐cultures imposed by managerialism (Horton and Lynch‐Wood 2020; Jones and Armit 2025; see also Bulutlar et al. 2025) which prioritise patient throughput over health, restrict contact time and allowed costs per patient ‐ the increasing trend of commodification of healthcare ‐ and may thereby compromise treatment quality and outcomes in certain cases (Timmis et al. 2026). Unnecessary bureaucracy in the healthcare sector not only generates frustration, lowers morale and increases stress in frontline healthcare workers, but additionally negatively impacts healthcare services in general, by reducing capacity and quality of healthcare (Husain 2024). Given the magnitude of the global healthcare enterprise and its unique role in promoting wellbeing and minimising human suffering, this impact is substantial.

Unnecessary bureaucracy similarly impacts social work by imposing operational constraints that increasingly restrict the autonomy of social workers and their ability to match measures to the needs of those for which they are responsible, and hence their ability to improve health and wellbeing (Pascoe et al. 2023). This has, for example, precipitated calls for change to the child‐protection system from one of excessive bureaucracy to a focus on children (Munro 2011), moving from a culture of compliance (Adler and Borys 1996) to a culture of learning, with more time and freedom for frontline workers to apply their expertise in assessing children's needs and providing timely and effective help.

Another relevant aspect is patient frustration and stress associated with navigation of healthcare bureaucracy. **“**They (Kyle and Frakt 2021) find that nearly one‐quarter of those surveyed report delayed or foregone care due to an administrative task. Figuring out which forms to fill out, which doctors their insurer will allow them to see, what will and will not actually be covered, as well as getting pre‐authorisations for covered care and arguing over bills, consumes patients' time, money, and emotional energy. Access to care, of course, also hinges on access to health insurance, which entails its own set of burdens, ranging from trying to figure out which plan will actually meet your needs for a reasonable cost to the own peculiar set of bureaucratic obstacles to accessing public health insurance like Medicaid.” (Herd and Moynihan 2021).

It is important to emphasise here that bureaucracy in healthcare is special, probably unique, in that physical and mental disorders can amplify negative impacts of unnecessary and overdimmensioned bureaucracy, and also impact those who are normally fit and able.

### 3.3 Smothering Creativity and Innovation

The ability of humanity to advance and solve the problems it faces is aided and promoted by acquisition of new knowledge and understanding through discoveries and innovation that translate discoveries into practical applications and solutions. Significant progress can occur when the not obvious is recognised, when connections are made between apparently unrelated things, when an entirely new method is developed and applied to pressing problems; that is, when original discoveries are made. Making original discoveries depends upon the creativity of researchers and innovators imaginatively exploring idea‐concept‐solution‐implementation space through independent/systems/lateral/critical thinking. Creativity is generally considered to be the ability to produce something that is both novel/original and valuable/useful/meaningful (Simonton 1999; Unsworth 2001; Hennessey and Amabile 2010; Ohly 2018). It is not a faculty that can be simply turned on or off, controlled, channelled, or scheduled for 16.15 on Friday afternoon, like a video conference (Unsworth 2001). Creativity involves risk‐taking. In essence, the conditions imposed by managerialism are the antithesis of those that nurture imagination, creativity and innovation, namely independence and free thinking, an exploratory questioning‐challenging environment in which critical and systems thinking, and scepticism of proposals and practices the values of which are not grounded in evidence, prevail (Unsworth 2001; Klikauer 2013; Ohly 2018; Robinson et al. 2025). For creativity to function, the mind has to be receptive to new insights/ideas/relationships. Creativity and receptivity/intellectual bandwidth may be blocked by routine attention‐intensive activities in the workplace and a risk‐aversion culture (e.g., Walter 2012; Husain 2024). But the rest of the time—in the shower, while jogging, while having a coffee/lunch with colleagues, deep reading, etc.—is available for creative thinking. Except that this is also blocked by frustration and anger that fills the mind when boring, unrelated tasks that seem to be entirely without value to anyone are imposed. *Unnecessary bureaucracy is a major impediment to the creativity that drives scientific progress and innovation*.

As stated in the Draghi Report (European Commission 2024), “we (*Europe*) claim to favour innovation, but we continue to add regulatory burdens onto European companies, which are especially costly for SMEs and self‐defeating for those in the digital sectors. More than half of SMEs in Europe flag regulatory obstacles and the administrative burden as their greatest challenge.” An independent review of UK research bureaucracy highlights the importance of high‐level actions that can ameliorate the bureaucratic burden to liberate time for creative research (GOV.UK 2021). This is encapsulated in the statement “No country bureaucratised its way to excellence” (Scott et al. 2022). Excessive bureaucracy imposes a major bottleneck on creative processes.

Collectively, bureaucratic impediments to the productivity of researchers, innovators and organisations that create and implement solutions to problems represent a global waste of talent, money and opportunity to achieve major improvements in the condition of humankind.

**Unnecessary bureaucracy is insidious and has wide ranging consequences for global productivity, sustainability and human wellbeing**

Though on occasion very noticeable in terms of impact and reaction, unnecessary bureaucracy is mostly incremental and accretive—new measures may be hardly noticed, are ignored or perceived with fatalism. Complaints about unnecessary bureaucracy are pervasive but usually restricted in scope and conducted in an atmosphere of helplessness: *it is simply that way, it is part of the job, there is nothing we can do, we should not complain because others are worse off* (and sometimes: *if we complain, our jobs*/*careers may be at risk*). Moreover, discussions usually revolve around the situation within an organisation—are siloed—not about the larger picture of global productivity, workplace harmony and human wellbeing. But, what is experienced in one workplace is replicated in many. Collectively, unnecessary bureaucracy is responsible for a significant global burden of reduced productivity, creativity, innovation and opportunity to address global and regional problems. Unnecessary bureaucracy is a force that hinders humankind's efforts to solve problems and attain sustainable development.

## 4 Imagination Infrastructure: The Missing Layer in Concepts Designed to Counter Excessive Bureaucracy

Imagination is the upstream resource from which discovery, problem‐solving and organisational renewal flow. Excess bureaucracy depletes this resource by (i) consuming frictionless hours with low‐value administrative demands, (ii) fragmenting attention through constant context switching, and (iii) converting curiosity time into compliance time.

*Imagination infrastructure* refers to the policies, spaces, tools, processes and protections that create, preserve and channel cognitive slack for creative work in science and other endeavours (see also Robinson et al. 2025). It can include tangible elements—protected time blocks, biophysical sensory‐enhancing technology, reflective or green spaces, sensory rooms, walk‐and‐talk routes, communal idea walls and rapid‐decision ‘green lanes’ for low‐risk activity—and intangible ones such as psychological safety, permission to try and fail, and proportionality rules that scale oversight with real rather than perceived risk. These conditions expand the bandwidth for novelty: more ideas expressed, tested and refined per unit time. Three design principles underpin strong imagination infrastructure:

- Slack by default—Protect uninterrupted time and a modest weekly quota for exploration and reflection. Treat cognitive and creative space as seriously as financial budgets.
- Proportionality by design—Match the weight of processes to the scale of risk. Replace routine micromanagement with trust‐based attestations and spot checks.
- Fast feedback—Regularly sense how rules shape creative capacity, and retire those that hinder rather than help.

Because infrastructure only matters if it lives in practice, organisations can lightly track a few health indicators—for example, the proportion of frictionless hours, the time from idea to first experiment, and simple feedback on administrative stress. These are not metrics for policing creativity but mirrors to keep conditions generative. In research and public‐service settings, imagination infrastructure is as vital as fiscal or IT infrastructure because it governs the throughput of novelty and renewal. Where it is weak, bureaucratic drag yields fewer ideas, slower iteration and delayed benefit. Where it is strong, organisations achieve more meaningful impact with the same resources—while nurturing wellbeing, curiosity and collective purpose rather than compliance.

If we designate productive creative output as **C**, then

C ∝ (Frictionless Hours) × (Psychological Safety) × (Autonomy)

Administrative burden reduces Frictionless Hours; surveillance‐style compliance reduces Psychological Safety; absolutist rules reduce Autonomy.

Policy lever: raise any one term (e.g., protected slack time, proportionate rules) and you increase C even if budgets stay flat.

## 5 Some of the Unacceptable Aspects of Unnecessary Bureaucratic Measures That Need to Be Addressed

Complaints about bureaucracy range far and wide, some specific to particular settings, but to seek solutions, it is important to identify and address generic root causes of frustration. These are some of those we consider to be the most important:

### 5.1 Lack of or Inadequate Convincing Justification for, or Demonstration of Inherent Merit of, Imposed Administrative Tasks

A major criticism of some tasks imposed by some administrations is a lack of obvious rational purpose, essentiality, and value to the enterprise. Most such tasks are not justified (or plausibly justified) to those affected, so frustration engendered by loss of assigned time for productivity is amplified by frustration of not seeing the sense of the task (some personal examples are provided for illustration in the Appendix 1). Even if justifications are provided, they may be unconvincing, such as:

- *We have no choice*. Often, when a new burdensome task is introduced, a justification is: *this is imposed upon us from above/outside, we have to impose it on you*. While this is sometimes true, it may also sometimes be an excuse because (a) it may be a recommendation, not an obligation, which constitutes misrepresentation, (b) it may embody a degree of flexibility in its interpretation which is not communicated to those affected when adopted.
- *It is the norm/widely adopted/state*‐*of‐the‐art*. This is the lemming argument, which obviously has no inherent validity since it ignores the necessity to demonstrate inherent merit. There are many examples of blindly following others—groupthink (Turner and Pratkanis 1998; Timmis et al. 2025)—that led to disaster (e.g., Janis 1982)—*doing it right* rather than *it being right to do* (see Berwick et al. 2017).
- For existing measures: *it has always worked well, is time‐tested and experience shows that it is the best for us—*the “habit” justification (see Slim 2020). Again, no objective reasons are given. Importantly, the issue of benchmarking, which is a vital element in justification, is deliberately and explicitly excluded from the discussion. Some bureaucratic measures may have previously made sense or been useful but now return little or no benefit for the considerable frustration they entrain.

**The need for within‐bureaucracy critical assessment and justification of measures**

To facilitate understanding and acceptability of measures, it is incumbent upon bureaucracies to convincingly explain to those affected why a particular measure is necessary and what benefit‐value its adoption/continued practice will achieve. Even unpopular measures that are seen to be beneficial will be accepted by the majority. The development of such explanations is also often a helpful reality check that may result in reappraisal/modification of the measure. Moreover, such justifications are basic human relations—the respect of others—making an effort to reduce unnecessary aggravation and discord. It is equally incumbent upon bureaucracies to resist/push back any proposed or imposed measures that obviously have little merit for their enterprise. Administrators are not robots: they have agency‐intelligence and minds of their own that are able to evaluate the utility and essentiality of imposed or recommended measures in the context of their own organisation. A bureaucratic measure without obvious value is a problem. Administrations have the choice of dealing with the problem themselves or passing it on to the workforce. The latter is easiest but obviously unethical and counterproductive. The former requires acceptance of responsibility, dedication, courage and the support of superiors, and is largely underpinned by a sense of contributing to the vision of the enterprise and being valued for doing so. It is a fundamental duty of administrations to object to and push back against measures that do not convince, and to shield the workforce from them.

### 5.2 Absolutism Instead of Proportionality: The Need to Relate Measures Imposed to Their Goal(s)

Often, a ‘problem’ (e.g., money laundering and financial corruption) is identified that requires a ‘solution’ (e.g., controls on money movement). The problem, while real and in urgent need of a solution, is however sometimes viewed as qualitative (black and white) rather than quantitative. A quantitative problem is usually best solved with a quantitative solution. An example of a constant irritant are the bureaucratic tasks scientists need to complete in order to receive reimbursement of expenses for travel undertaken in the interest of the enterprise. Common sense dictates that the greatest *effort* to frustrate money laundering must be exerted at the source of the greatest *quantity* of laundering (e.g., see https://home‐affairs.ec.europa.eu/policies/internal‐security/corruption_en, where numbers approaching € 1 trillion are discussed). The black‐white approach that is sometimes adopted imposes essentially the same regulatory controls to frustrate improper transactions of < € 1 and > € 1 million, an example of Parkinson's Law of Triviality (https://www.economist.com/news/1955/11/19/parkinsons‐law). Clearly, heavy administrative burdens for small sums (a) have essentially no effect on the problem of money laundering and financial corruption, so are not fit for purpose, and (b) waste significant time = money, so the net benefit is usually negative (see Appendix 1). Why is this? Three possible reasons among many are: (a) simplicity: a one‐hat‐fits‐all process is simpler to administer, (b) *passing the buck*: it is easier for (often, but not always, external) higher levels of administration to delegate a problem to lower levels and convince themselves that they have thereby done their duty, which is obviously not the case because effectively addressing the problem requires high level action, e.g., in the case of money laundering‐financial corruption, at the level of organised crime and corruption at the highest levels of government (Katanich 2024; https://www.transparency.org/en/cpi/2023). Such action obviously requires courage and considerable resources and faces resistance by vested interests, (c) envy? The lives of ‘producers/deliverers’ may sometimes seem to others more important/exciting. In particular, the travel carried out in the service/interest of the enterprise, sometimes to attractive destinations, may seem to some more like a holiday than work. But bureaucracy does not exist for the exercise of simplicity, *passing the buck* or envy: it is duty‐bound to rise above and deal with issues with effectiveness, agnosticism and above all in a manner that leads to the best overall value for the enterprise, the value of which employee motivation is not inconsiderable (Hinsz 2008).

Proportionality‐scaling is required in such issues, with no administrative burden for small sums. What constitutes a small sum should not be decided *ad hoc*, but be evidence‐based using cost: benefit/return on investment analysis that includes in the modelling the cost of time lost to the bureaucratic tasks *in toto* (i.e., investment of time by producers, and by bureaucrats checking the information provided by the producers). Regulations and systems must be proportionate to the need, e.g., in reimbursement of travel expenses proportionate to the magnitude of the money involved and the level of risk. In any case, for modest sums, an honesty system coupled with spot checks is known to be both highly effective at dissuading dishonest practices and the least burdensome for everyone concerned (Baldwin et al. 2012).

### 5.3 Absolutism Instead of Proportionality: The Bureaucracy of Safety and the Need to Relate a Risk Under Consideration to Other Risks

Probably the most extreme example of proportionality vs. absolutism is in the domain of safety. When developing safety regulations, it is not unusual to consider the case in question in isolation: if something negative can happen in a particular activity, it must be prohibited. But this is “silo thinking” when in fact risks need to be considered in context—relativised in relation to other relevant risks, such as the risks of accidents while driving, health risks associated with smoking, the increased risk of skin cancer caused by sunbathing, the inherent health risks of childbirth, etc. In all these cases, the risks are known and more or less quantified, and accepted: there is no prohibition. Humans, like all organisms, engage in risky behaviour—it is nature. While it is undoubtedly prudent to promote behavioural changes to reduce known risks, the issue of personal freedom of choice must also be considered, and efforts to discourage risks should also take into account and seek symmetry with current efforts to discourage existing known risks: measures need to be based on local expertise, common sense, plausibility and what measures are in place for other basic risks.

The most extreme version of irrational development of safety regulations is that based on/determined by *What if?* For example, genetically‐modified organisms have been around for 50 years. They have brought incalculable benefits to humanity, including vaccines that prevented millions of unnecessary deaths. Nevertheless, there still exists a fundamentalist movement against the use of these organisms based upon *what if? What if* some harm is detected after 60 years… 100… 1 million? Many such discussions take place in a framework based upon a requirement of zero risk. But zero risk does not exist in nature. Bureaucracies should never enter the illusory framework of zero risk.

### 5.4 Inconsistency

Another aspect of diversity in administrations is the issue of getting different responses from different people. People are often crudely classified into problem solvers and problem creators, though there is obviously a range of intermediates/grey areas. Problem creators bear considerable responsibility for the bad reputation of bureaucracies and, when confronted with the question: *why do you refuse when your colleague agreed last week*? inevitably come up with the justification: *that is the rule/law*, explicitly implicating the other colleague in breaking rules/laws, rather than taking on board the possibility that the colleague may actually be doing a better job in making pragmatic sensible decisions using the degree of freedom allowed by the system.

Constraining negative behaviour of this type is essential in order to avoid reducing engagement, motivation, workforce morale, team spirit, and performance. Having open discussions about it will undoubtedly help and lead to a dampening of obstructive behaviour. However, open discussions face the problems that those affected may believe that problem solvers may be sanctioned within the bureaucracy, and the problem solvers may not want to confront the problem creators and provoke added strain in group dynamics (Murthy 2022). Nevertheless, while recognising such challenges, diversity of decisions, pragmatism and contributing to a healthy organisational culture must be openly discussed. In such discussions it may be useful to pose the question: *how would the problem solvers' response negatively impact the success of the organisation*?

### 5.5 Discriminatory Nature of Some Bureaucratic Measures

While bureaucratic procedures can seriously stress the fit and able (Graeber 2015), they disproportionately disadvantage the old, frail, and those with disabilities (Kohl 2020; Pianosi et al. 2023), a segment of the population that is increasing and in a Canadian study published in 2017 constituted 22% of the working age population (Hebert et al. 2024; see also WHO 2011). Particularly vulnerable are those with deteriorating cognitive functions, people who experience difficulty handling complex issues or understanding official language, and people alone and lacking support networks, who may have more difficulty than usual navigating regulations and who may be criticised and penalised for mistakes/incompleteness. The old and frail may also have difficulty with digital operations; this may include not only those who did not grow up in the digital world, but also those who did, once their cognitive functions start to decline (Hachouch et al. 2025). This can constitute a significant stress factor and may exaggerate existing mental health issues, including pre‐dementia conditions, impacting quality and duration of life. Such situations can constitute a particularly insidious form of discrimination, in extreme cases, even abuse. The less educated and the illiterate or indigenous peoples may be particularly vulnerable.

While the bureaucratic language and formulations of forms, and the necessity of all information requested, are often unclear to many people, those whose mother tongue is not the same as that used in the form experience greater difficulty and stress, which goes counter to extolling the advantages of a multi‐cultural society. While it is neither possible nor desirable to provide all forms in all possible languages, a much wider appreciation of and sensitivity to the issue, coupled with serious concerted efforts to simplify forms and their language, would certainly ameliorate the problem. The desire to cover all eventualities ordinarily causes more problems than it solves and should be discouraged (see the issue of proportionality below). Another, related, issue is inadequate quality control in the updating of forms and information. Forms which originally may well have been coherent, consistent and readily understood sometimes lose these qualities over time through *ad hoc* revisions that fail to incorporate adequate quality control (“When you fill out the form, and none of the listed options fits your circumstances, you must adapt yourself to the form, rather than the form adapting to you”. Harari 2024).

Failure to adapt bureaucratic measures to those who experience difficulty in dealing with them is an example of cost externalisation (see below), namely externalising bureaucratic time‐effort costs which translate into difficulties borne by others, in this case, the more vulnerable members of society.

In addition, online tools are promoted as time‐saving and agnostic (not subject to diversity of human opinions and decisions), which can be true. Unfortunately, many software programmes are poorly conceived and constructed, and some can readily lead into perpetual unproductive loops. Navigation of some web programmes is difficult for some and impossible for others. The failure of some bureaucracies to carry out adequate quality control of their programmes is also a cost externalisation measure and contributes to workplace frustration and anti‐bureaucracy prejudices.

### 5.6 The Special Case of Administrative Burden on Those Already Under Severe Stress

Certain individuals under certain circumstances experience severe stress. A typical example is the shock and grief over the loss of a loved one, but there is a wide range of causes including acute disease, financial strain and abuse. In many cases, the severe stress is compounded by administrative burden, whether it be the multitude of procedures dealing with a death (Herd and Moynihan 2018), payment and insurance related to a health disorder or social security claims (Kohl 2020), negotiation with companies and authorities as a result of inability to pay bills, or interacting with authorities to deal with abuse. While some bureaucratic procedures are unavoidable, and in some instances help may be available, in general the underlying circumstances represent significantly increased vulnerability to bureaucratic harm. In most cases, it is likely that these administrative measures were conceived without regard for the condition of the persons at the sharp end, i.e., without consideration of duty of care, Health in All Policies and the need to respect human dignity. This may constitute structural violence (Galtung 1993). There is a serious need for an overhaul of such procedures and a pruning back of those that are not essential.

### 5.7 Cost Externalisation

Externalisation of costs is costs of production‐activity that someone else pays (https://www.imf.org/external/pubs/ft/fandd/basics/38‐externalities.htm). The classic example of cost externalisation is an industrial process that pollutes the environment, the remediation cost of which is borne by the taxpayer and not factored into the costs of production and hence the price of the product marketed by the industry. Externalised costs can be huge: it has been estimated that externalised costs of the food industry can amount to $12 trillion annually (https://www.fao.org/newsroom/detail/SOFA2024‐8‐trillion‐in‐annual‐hidden‐health‐costs/en), of which some 9 trillion relates to health costs incurred, and 3 trillion relates to ecological damage (https://www.nature.com/articles/d41586‐019‐03117‐y). Cost externalisation usually refers to monetary‐economic costs, but can apply equally well to other, non‐monetary resources, such as health, quality of life and leisure, education‐career, nutrition, etc. (Timmis et al. 2025).

The example cited above of poorly designed/constructed forms and online tools whose use is obligatory is the externalisation of the cost of quality control that translates to the cost of unnecessary time and frustration borne by the user.

Another example results from the growth of managerialism (see below) in academic institutions. A career within academia is generally based in part or entirely on simple metrics like the number of publications and the impact factors of the journals in which they appear, and the level of success in obtaining research grants and the “overheads”—discretionary funds—that these may bring to the institution. However, in addition to carrying out and publishing research, academics typically have a host of other responsibilities that may contribute less or not at all to career advancement, such as teaching in its many forms, including examining and advising students, as well as outreach activities, training and mentoring early‐career researchers/educators, reading and assessing theses and reports, contributing to the running of the academic centre, research group management, development of research grant proposals, management of grants and writing reports, peer review of grant applications and papers submitted for publication to research journals by others, being members of committees at all levels, etc. Academics may also be heavily involved in advising external organisations, shaping government policy, as well as establishing spin‐out companies. Clinical academics often have additional duties, such as patient care, managing hospital diagnostic laboratories and result reporting, and so on. There is a serious disconnect between the broad spectrum of essential academic duties and the narrow spectrum of achievements that form the basis of academic career advancement, although there is some push‐back against this asymmetry (https://sfdora.org/; Woolston 2021; Jonjić et al. 2026).

An academic career, if it is to progress successfully, is often high octane, high stress and closer to 60 h than 40 h per week, often involving work both in the workplace and at home. A decision to adopt a healthier work‐life balance (https://en.wikipedia.org/wiki/Work–life_balance) can lead to lower research outputs, less impressive metrics and hence weaker competitiveness in the workplace, and be detrimental to career progression, although the lower workload is in part compensated by the increased creativity and autonomy.

Compounding the problem of time dedicated to tasks that may not count towards career advancement is an ever‐increasing raft of bureaucratic tasks that either add to the workload or reduce time available for tasks that do count towards career advancement. Bureaucratic tasks that are non‐essential seriously restrict autonomy and hinder career progression (Balzano et al. 2025). While academic duties that may not contribute to career advancement are nevertheless essential elements of academic life and, for the most part, provide stimulation and satisfaction, unnecessary bureaucracy is a significant source of frustration that magnifies this stress and degrades quality of life and family‐friend cohesion. Managerialism practices in academia that base career advancement on a few simple metrics, rather than assessment of productivity‐performance‐value of all relevant career parameters, increasingly introduce bureaucratic measures of questionable value and externalise these personal burdens.

Cost externalisation is facilitated by ‘silo thinking’—consideration of individual issues that in reality are part of a whole—and hindered by systems thinking (Timmis et al. 2025). There is an urgent need to expose and comprehend the consequences of cost externalisation wherever it occurs and to take steps to prevent it and compensate for it.

### 5.8 The Harmful Collection of Personal Data

Cyberattacks and the theft of personal data and its use for diverse criminal activities, especially by organised crime, are a major problem facing society. This is made easy—in fact promoted—by the collection of all manner of personal information by many different organisations, including bureaucracies, which, given the widely appreciated fact that most of their databases are readily hacked, are culpable in the success of this lucrative enterprise. These days, filling in almost any sort of form—even for online purchases—inevitably involves the provision of personal data. Forms querying personal data often have specific questions decorated with a star, indicating that such information is obligatory and that progress in form completion will not be possible without its provision. In many cases, there is no obvious justification for such a requirement, and it may be entirely unclear why some of the data is even requested.

Cyberattacks can not only result in the transfer of personal data into the criminal ecosystem and the potential harm this represents, but also the frustrating need to change passwords, the anxiety and stress related to possible criminal exploitation of stolen data and, when this does occur, a potentially transformative change in financial security that can engender a degradation in quality of life and associated worry and stress. These, in turn, may trigger new mental issues or deterioration of existing ones, which can lead to erosion of relationships within and between social and support networks of families and friends. Cyberattacks are a contributor to unnecessary suffering, as indeed is any act that facilitates them. Because hacking skills are generally better than most data protection systems, the collection of unnecessary personal data is a participatory act in any harm caused by cyberattack.

What is the motivation for the unjustified comprehensive collection of personal data? There are a number of official reasons, including authentication, but most will fall down when confronted with the question: what would be the consequence of non‐provision of this piece of my personal information? The real issues are: (a) profit: use for customer data and preference analysis exercises and/or sharing with other organisations for the same or different purposes (revealed by a question/statement or small print stating that data will be shared with other organisations), (b) the discredited question: *what if?* It does not have an immediate relevance, but might conceivably be useful in the future, and better to collect it now than be bothered with another task later and (c) everyone else does it, so it must be useful/right/profitable.

Unnecessary and unjustified collection of personal data is an important component of unnecessary bureaucracy and yet another example of cost externalisation, in this case, externalisation of the costs of loss of personal data by transfer from those collecting to those from whom the data is collected. It contravenes the principle of duty of care and Health in All Policies. Things would undoubtedly change for the better if this type of cost were not externalised, and the collectors of such data would be legally liable for any loss of data from their systems.

**The collection of personal data without convincing justification must be constrained**

The collection of personal data by bureaucracies must be subject to strict rules based on convincingly justified necessity and to independent oversight. The need to obtain ethical approval for research involving human subjects might be a model for obtaining personal data because it is based on similar principles, namely protection against harm of individuals and of confidentiality of their data. A breach of rules should have legal consequences. Bureaucracies, especially those of civil services, must take the lead in this. This is also an issue of duty of care and Health in All Policies.

## 6 An Analogy With Obesity

Essential bureaucracy is a vital force that steers and supports organisations and their activities, thereby contributing to their success. Excessive bureaucracy is often indicative of being overweight, a “silent disease” somewhat analogous to obesity. Obesity is often a slowly developing condition that impairs diverse metabolic processes, resulting *inter alia* in cardiovascular disease, diabetes, cancers and neurological, respiratory and digestive disorders. The global health and economic impact of obesity is both huge and increasing: obesity is considered to be an epidemic and in 2022 affected one in eight people −1 billion—and in 2021 was responsible for almost 4 million deaths worldwide. It is estimated that the global economic costs of obesity will be $3 trillion by 2030 (https://www.who.int/news‐room/fact‐sheets/detail/obesity‐and‐overweight). Unnecessary bureaucracy is a growing disorder that entails “metabolic disease” in organisations and high global costs. Crucially, like obesity, the crisis of unnecessary bureaucracy is largely preventable.

## 7 Some Potential Causes of Excessive Bureaucracy

### 7.1 The Rise of Managerialism and the Seemingly Never‐Ending Increase in Bureaucratic Tasks

Managerialism is an ideology that lauds restricted context cost–benefit analysis, top‐down targets, and data‐driven measurement, often at the expense of system‐wide effectiveness, autonomy, legitimacy, and equity (Tsui and Cheung 2004; Burton and van den Broek 2009; Alvesson 2013; Klikauer 2013; Al Mahameed et al. 2024). The time spent by almost everyone on bureaucratic issues—especially form filling, reporting, the search for supporting documents, related meetings and discussions, and lengthy periods of waiting—just seems to increase inexorably—the *Law of Multiplication of Work* (https://www.economist.com/news/1955/11/19/parkinsons‐law; Hood and Dixon 2015). In so doing, these tasks increasingly occupy and replace time designated for the primary tasks of individuals and groups of an enterprise that determine its productivity, competitiveness, and success. This poses the question: at what point will bureaucratic tasks replace primary tasks to the extent that the former becomes a hindrance rather than a help? Have some organisations already reached it? What fraction of our lives will be dominated by bureaucratic tasks in 10 years, if there is no concerted action to cap it at a reasonable level (Klimek et al. 2008; https://www.economist.com/news/1955/11/19/parkinsons‐law)? Concerted push‐back against measures that seem to have little merit is vital (https://www.timeshighereducation.com/news/managerialist‐overreach‐biggest‐problem‐academics). It is not for nothing that the question has been posed: at what point will an organisation/civilisation collapse under the weight of excessive bureaucracy (see, e.g., Graeber 2015; Tainter 1988).

### 7.2 A Privilege Nexus of Permanent Positions–Privileged Growth: A Lack of Organisational Symmetry

Whereas many organisations, particularly commercial enterprises, grow and contract according to strategic needs and prevailing economic circumstances, and staff can be shed when necessary (which obviously provides an opportunity to acquire new skills and increase efficiency), bureaucracies are usually protected from such contractions, at least in civil service ecosystems and the like (https://www.britannica.com/topic/bureaucracy). Some bureaucracies may grow in size without any obvious reason, even when the size of the enterprise itself is not growing, or even when it may be contracting (https://www.economist.com/news/1955/11/19/parkinsons‐law). In some cases, bureaucracies can thereby expand out of proportion to the total workforce and the needs of the enterprise, reducing resources that would otherwise benefit the activities of the enterprise, and hence become a financial drain on the enterprise. A commonly‐held view is that career progression in bureaucracies more involves increasing the number of people supervised—*The Law of Multiplication of Subordinates* (e.g., https://www.economist.com/news/1955/11/19/parkinsons‐law; https://www.bbc.com/worklife/article/20191107‐the‐law‐that‐explains‐why‐you‐cant‐get‐anything‐done)—than the normal factors leading to promotion, such as increased productivity, success in reaching targets, creativity, problem solving, etc.

The consequence is that, in some organisations, there may be an incentive for their bureaucracies to expand uncoupled from their contributions to productivity (“administrative bloat” Yang and Grenier 2025; see also Slim 2020). According to this narrative, expansion is justified by adopting and applying more and more bureaucratic tasks—*The Law of Multiplication of Work* (https://www.economist.com/news/1955/11/19/parkinsons‐law)—some of which take up valuable time of other members of the workforce (in some cases, some of which may be of dubious value to the enterprise), or by exaggerating the workloads of existing tasks leading to poor performance, slow reactions, etc. (see Parkinson's Law above). *The conflict of interest that arises when considering the adoption of new measures that will engender new work requiring additional personnel is evident.* While these perceptions may be unwarranted in some cases, it is important to counter them with transparent mechanisms that prevent their development and a need to base career development on the quality and efficiency of work, and its contribution to the excellence and productivity of the enterprise.

In many organisations, and in civil services in particular, positions in bureaucracies are essentially permanent. This has been historically justified by the need to ensure the continuity that is essential to efficiency, but an element of loyalty to the leadership of the organisation may also exist implicitly or, in some cases, explicitly by means of an oath (https://www.legco.gov.hk/research‐publications/english/2021rt03‐oath‐taking‐requirements‐for‐public‐officers‐and‐the‐consequences‐of‐non‐compliance‐in‐selected‐places‐20201224‐e.pdf). But continuity can no longer be a justification, since bureaucratic procedures are standardised, digital records enable almost immediate access to prior information, and experience is a quality equally valued in many other members of an organisation. Importantly, the diversity of technical skills needed by bureaucracies has grown significantly in recent years. If all members have permanent positions, it can be a challenge to avoid ending up oversupplied in traditional skills and undersupplied in new skills, resulting in unnecessary bottlenecks in some areas and excess capacity in others, both of which cause unnecessary wastage of financial and personnel resources. The need for new skills is also a frequent argument to expand the personnel of bureaucracies, even though they may already be overstaffed and some members underworked. Permanent positions for all or most members of a bureaucracy sabotage the essential need for flexibility in a changing world.

This situation may starkly contrast with employment possibilities and permanent posts for frontline staff, as exemplified by young academics. These typically have 6–10 years of postgraduate training and experience, even longer for clinical academics. Despite this, many have serious difficulty in obtaining an academic post offering a promising career trajectory, even for those willing to consider possibilities in other countries, and most spend several more years in posts of limited duration before a more stable one becomes available, if indeed it does.

There is no need to treat members of the bureaucracy differently from other members of an organisation; such organisational asymmetry constitutes discrimination and a cause of resentment against bureaucracy. Moreover, this form of discrimination has a serious potential consequence: permanence of all/most members of a particular unit in an enterprise, in contrast to members of other units, can convey a sense of entitlement and superiority, which can in turn lead to arrogance. In some cases, the knowledge that a position is permanent can induce a belief of invulnerability and, in those disposed to it, lower restraint of bad behaviour. Arrogance and bad behaviour exist everywhere, so they are nothing special. What is special about bureaucracies is that their individual members can create unwarranted personal power by simply blocking, delaying, requiring completion of further tasks, or creating problems relating to their responsibility of delivering essential goods or services that others absolutely depend upon (https://www.currentaffairs.org/news/2020/12/how‐bad‐bureaucracy‐sabotages‐democracy). For people so disposed, permanence provides more opportunities for abuse of power than those existing for their non‐permanent equivalents. If not vigorously countered by the leadership, this can lead to a toxic work culture.

There is a sense among some that, as a result of the privilege nexus, some bureaucracies have uncoupled their work from the needs of the organisation (https://www.cato.org/sites/cato.org/files/pubs/pdf/fpb003.pdf). The solution to the problem of the privilege nexus is clearly the removal of this privilege and insistence on the culture of professional service.

### 7.3 Not Appreciating the Importance of Change for Progress

The traditional view of bureaucracy is that it creates order and prevents chaos. However, progress usually occurs through discovery and innovation, the most significant of which can be “disruptive”: they perturb the existing order and eventually lead to a new order. In resisting changes to established order—in preventing what they perceive as chaos—bureaucracies may create chaos and become impediments to progress (Abbey 2021). Indeed, some bureaucrats see their *raison d'être* in the control and restraint of those who discover. This reminds us of the Pareto Principle: perhaps 20% of the population create and 80% do not, including some bureaucrats who strive to resist creativity by generating unnecessary bureaucracy. Perhaps paradoxically, disruptive discoveries that initially create disorder may be resisted by bureaucratic disorder. If this resistance is great enough, the organisation will no longer be fit for purpose and itself become unnecessary; it risks collapse under the weight of its bureaucracy: the *bureaucracy tipping point*.

For bureaucracies to align with discoveries and progress, and support changes that are essential for the healthy development of their organisations, it is essential that they have a built‐in mechanism that promotes their own change in step with organisational needs. This is the Panta Rhei Principle of Heraclitus (https://plato.stanford.edu/entries/heraclitus/?utm_source=chatgpt.com)—*everything flows* (all things are in flux and stability is an illusion; change is the only constant) which applies not only to actions but also to the structures of bureaucracies, and the need to nurture the talents of the young so that they can promote positive change.

### 7.4 Changes Made for the Benefit of the Administration

Bureaucratic measures must serve the interests of the enterprise as a whole. When a new bureaucratic measure is or appears to be beneficial in the restricted context of the administration, but increases the workload of others, its overall benefit may be questionable. It may be considered to be the bureaucratic equivalent of cost externalisation. It is therefore vital to justify new measures with an objective and transparent system‐wide cost–benefit analysis that factors in all relevant elements and that is subjected to scrutiny by all affected.

### 7.5 Fragmentation of Responsibilities

The tendency for increases in size of bureaucratic units often leads to the creation of new responsibilities/“portfolios” (reorganisations are sometimes justified by purported increases in efficiency but sometimes are simply career development exercises, sometimes involving the creative invention of impressive job titles that often fail to convey underlying job responsibilities), but this may result in fragmentation of responsibilities and lowered efficiencies. “Turf battles” may then develop and frustrate solutions being found for important problems (e.g., see Abbey 2021).

## 8 Why Has There so Far Been Limited Pushback Against Unnecessary Bureaucracy?

Unnecessary bureaucracy is a highly charged issue that often triggers emotional outbursts when broached. Curiously, and irrationally, this strength of feeling rarely translates into any meaningful action; there is a collective sense of helplessness against a pervasive, all‐powerful force. In seeking a solution strategy to constrain unnecessary bureaucracy, it is essential to consider why there is so little pushback and effective reform, and how this situation may be changed. Some potential reasons are as follows:

### 8.1 A Primordial Need of Humans for Order in Life

Hunter‐gatherers had order imposed by the vital tasks of survival, so they did not need a bureaucracy. Today, survival is not an issue for most people, and order is sought through bureaucracy; there seems to be an elemental need in many people to belong to and have a role in a rule‐based group/organisation/network. Thus, bureaucracy is created and supported by most people as part of a need to justify their existence. Without bureaucracy, society would perhaps be crowded with people suffering from burnout. Bureaucracy is thus essential for a number of reasons.

### 8.2 A Controlling Narrative Coupled With Failure to Distinguish Essential From Non‐Essential Bureaucracy

A frequently repeated conclusion in discourses on bureaucracy is that it is an important element in the collapse of civilisations, societies, businesses and organisations because bureaucratic expansion and over‐reach eventually replace activities vital to survival of the system or reduce it to the point where collapse is inevitable—*the bureaucratic tipping point*. However, the reason why bureaucracy was/is allowed to develop in this way is rarely discussed. One potential explanation is provided by Harari in his books “Sapiens: a brief History of Humankind” (Harari 2014) and especially “Homo Deus” (Harari 2015), in which he argues that humankind distinguishes itself from other members of the animal kingdom by its imagination and the susceptibility of collective imagination to hijacking by a few individuals who create a narrative that is adopted by the majority within a group: groupthink (Alvesson 2013; Timmis et al. 2025). Challenging such narratives is often difficult and mostly too late to save the enterprise.

Narratives rule human behaviour. While those grounded in evidence, such as “vaccines save lives” are beneficial, those that are not, such as “vaccines cause autism” can be harmful. The importance of bureaucracy for order and the good functioning of society, and managerialism ideology (Klikauer 2013), are one such current narrative. However, essential (beneficial) and non‐essential (distracting/burdensome) bureaucracy are not distinguished in this narrative (see also Adler and Borys 1996). One possible reason for a lack of effective push‐back against unnecessary bureaucracy may thus be a failure to distinguish between it and necessary/desirable bureaucracy. Where the difference is seen, there may be an unwillingness to challenge the generally accepted narrative, the groupthink.

### 8.3 Dependencies

Some bureaucracies, especially those in the context of civil service, are monopolies—there is only one office that can supply a passport, residence permit, reimbursement of travel expenses, etc.—there is a lack of choice and hence a lack of competition, a lack of an alternative if unsatisfactory service is provided. This creates dependency, a relationship that is vulnerable to abuse. Dependencies also exist within commercial and non‐profit organisations because the bureaucracy has a monopoly on all manner of goods and services, some of which may be facultative (e.g., permission to take time off for a wedding or funeral; awarding of an annual bonus; even promotion), so frontline staff may seek to avoid actions that may be perceived as antagonistic, to avoid “sticking out from the crowd” and potential sanctions (Adler and Borys 1996; Murthy 2022).

### 8.4 The “Devil You Know”

Things can always get worse and changing an unsatisfactory system may not achieve an improvement.

### 8.5 The Why? Question

When the utility of a new measure seems questionable, the obvious thing to do is to pose the question “why?”. But, as we have noted elsewhere (Timmis et al. 2025), although young children perpetually ask questions, this habit drastically diminishes with age as questioning is replaced by the need to deliver answers. This begins in school and continues in professional life. Moreover, asking questions may be actively discouraged, even when the intention is to improve a process or solution, and seen as disruptive or disrespectful. Those who would ask questions may well think that they do so at the peril of their career. Where this occurs, it is an issue of controlling the narrative, which is clearly counterproductive to positive change. There is an urgent need for a paradigm change in facing unnecessary bureaucracy: from reluctant acceptance to proactive engagement through questioning and demanding explanations that convince.

### 8.6 Attempts to Reform Bureaucracies

Attempts to reform bureaucracies are nothing new, as exemplified by the National Partnership for Reinventing Government in the 90s (Gore 1993) and more recent initiatives, such as “Zero bureaucracy” (https://mof.gov.ae/en/about‐ministry/mof‐initiatives/zero‐bureaucracy/) and “busting bureaucracy” in the health and care system in England (GOV.UK 2020). However, although such attempts may result in some changes for the better of individual bureaucracies, there is no general improvement and if anything excessive bureaucracy is increasing. Most attempts at reform address symptoms, mostly specific to a particular type of bureaucracy such as that of government (https://mof.gov.ae/en/about‐ministry/mof‐initiatives/zero‐bureaucracy/), healthcare or research, rather than considering generic causes of problems and proposing generic mechanisms aimed at effecting wide‐ranging fundamental change in behaviour and culture. Crucially, even when measures taken have a positive effect, such effects tend to diminish over time (https://www.governing.com/archive/gov‐reinventing‐government‐book.html). It is our thesis that effective reform requires solution strategies that not only address fundamental causes of excessive bureaucracy but also involve sustained monitoring of and corrections to policies and activities.

## 9 Lessons From Cellular Systems

Although it may seem far‐fetched to compare human activities with those of other systems, it seems worthwhile to us as microbiologists to ask whether microbial communities, and indeed individual cells of all organisms, which also have their bureaucracies—their diverse and multilayered regulatory systems that control and are responsible for order in metabolism and ecophysiological processes and behaviour—have characteristics that can be instructive in the task of reducing unnecessary bureaucracy (see Armstrong 2025, for a comprehensive overview of what microbes can teach humankind).

Chaos generally results from a lack of effective organisation and control, and this is certainly observed in biological systems when controls break down, as in disease and death of a cell or an organism, or disruption of the web of interactions of a community, caused, for example by an abrupt change in pH or oxygenation. However, when a cell or organism dies and its metabolism becomes chaotic, it is recycled by other cells or organisms which themselves are subject to organisation and controls that ensure order at a higher level: the dead cell simply becomes a resource for a larger system. Similarly, a community that is disrupted may morph into another that is stable and functional. Thus, although chaos can readily and regularly occur within discrete compartments of a system, overall order is maintained at higher levels (this may also be true over very different scales: the entire biosphere may well become chaotic as planetary boundaries and global warming thresholds are exceeded, but celestial order will be maintained).

Cellular and microbial community orchestration may offer important lessons for human bureaucracies. Firstly, cell‐community *bureaucracies* also exist to ensure that the best productivity (e.g., in terms of growth, production of a metabolite (e.g., hormone), etc.) is achieved under the prevailing conditions. Secondly, in general, they are highly responsive to change and challenges, such that the cell‐community rapidly reacts to changes in the environment and adapts to achieve maximal efficiency, sometimes enabled by recruiting new genes from other organisms via lateral gene transfer/by recruiting new organisms. Thirdly, and especially importantly, the proportions of cellular components/community members are finely tuned to the needs of the system; imbalances of components and unnecessary wastage of resources—of both actors and material and energetic inputs—that arise are quickly detected and corrected. Efficient quality control systems promptly detect and correct errors. Importantly, microbial systems lack inter alia individual ‘personalities’ and egos of players that ‘colour’ inherent abilities and influence outputs, labour relations, organisations, humanitarian and societal codes. They do not have gurus and consultants and do not adopt new fads, fashions, or mantras. They have evolved for success through selection.

However, while cells are evolved units of selection whose internal regulatory systems have been shaped to maintain coherence, microbial communities are not generally organisms in this sense. Their apparent order typically arises because certain configurations of interacting types are more persistent under given conditions. When a configuration becomes unstable, it may be replaced by another. Order at the higher level is therefore maintained, but often by turnover rather than inherited coordination. When communities endure over time, and when genes move readily among members via horizontal gene transfer, functions that support stable interactions can spread. In this way, something that resembles optimisation can emerge, not because the community is a unit of selection with collective intent, but because configurations that work tend to last.

This stands in contrast to human bureaucracies. If anything, bureaucracies are *even less* likely to be shaped by system‐level optimisation. They are rarely selected as integrated wholes. Instead, their structures tend to reflect individual, departmental, or political interests that can persist independently of performance. Layers accumulate because they serve someone, not necessarily the functioning of the system. Human bureaucracies often lack effective monitoring systems and pressure for corrective actions to optimise efficiency. Redundancy and permanence of roles, together with procedural accretion, can continue unchecked, even when they inhibit activity.

Of course, the analogy must not be taken too far. Cells and microbial communities do not have moral obligations or commitments to fairness and care, which are essential in human institutions. But the basic lesson holds: coherence and adaptability depend on proportionate, responsive regulation rather than the accumulation of layers whose persistence is justified only by their own inertia.

## 10 A Solution Strategy: A Bureaucracy Charter and Its Implementation: Principles to Govern Bureaucracies and Their Activities

Having identified what we consider to constitute some of the main problems and causes of unnecessary bureaucracy, we propose here a solution strategy based upon a Bureaucracy Charter and a mechanism to ensure its implementation.

A Charter, such as the United Nations Charter (https://www.un.org/en/about‐us/un‐charter/full‐text), the Charter of Fundamental Rights of the European Union (https://www.europarl.europa.eu/charter/pdf/text_en.pdf), and the Alberta Health Charter (https://www.alberta.ca/alberta‐health‐charter), *inter alia* sets out the purpose of an organisation or the key elements of a policy, the principles according to which it operates, its functions and procedures, and how it is governed and regulated. We propose here some key elements of a Bureaucracy Charter:

### 10.1 Function

Bureaucracies exist to maximise the good functioning, health and success of their organisation. They have no self‐purpose and only exist to *serve* the systems of which they are part.

### 10.2 Accountability

Activities of bureaucracies and their members need to be fully transparent, convincingly justified to those affected, and accountable.

### 10.3 Stakeholder Consultation

Appropriate consultation of stakeholders must take place before new measures are introduced; periodic consultation of stakeholders should take place about the appropriateness and efficacy of existing practices.

### 10.4 A Focus of Effort to Develop and Nurture a Healthy Organisation Ecosystem

Workplaces characterised by team spirit, community purpose, engagement and satisfaction in collective achievement are generally healthy ecosystems (see Faragher et al. 2005) that significantly contribute to the productivity and success of the organisation. However, attitude positivity is not a given and requires considerable and sustained investment of effort to create and maintain, especially by bureaucracies, focusing on a healthy corporate culture (https://www.investopedia.com/terms/c/corporate‐culture.asp) and symmetrical partnerships with other units of the enterprise. Equally importantly, well‐functioning bureaucracies and their members should be appreciated, acknowledged and rewarded for good service to the organisation and the individuals of its workforce.

### 10.5 Duty of Care

Bureaucracies have a *duty of care*. But, because of the nature of their role, this is not just restricted to the members of the bureaucracy itself, but also and especially extends to the people over whom they have influence. Importantly, where their activities extend beyond their organisation, such as those working with the general public or sections of it, there exists a duty of care also for the other people with whom they deal. The tasks they impose upon others and the services they provide to others must therefore be implemented within a framework of duty of care. That is: their policies and activities must minimise frustration and stress and certainly not result in harm. Perpetuation of existing unnecessary procedures/tasks, and introduction of new unnecessary tasks and procedures, etc., are in direct conflict with the obligations of duty of care. Failure to exercise duty of care can be discriminatory in nature. All bureaucratic measures should be subjected to a duty of care assessment.

### 10.6 Health in All Policies

*Health in All Policies* (HiAP) aims to avoid or at least minimise potential harmful health impacts of a wide range of human activities by being an integral component of policy development (https://eurohealthobservatory.who.int/publications/m/health‐in‐all‐policies‐prospects‐and‐potentials; https://eurohealthobservatory.who.int/publications/m/health‐in‐all‐policies‐seizing‐opportunities‐implementing‐policies; https://iris.who.int/bitstream/handle/10665/112636/9789241506908_eng.pdf?sequence=1#:~:text=Health%20in%20All%20Policies%20is,population%20health%20and%20health%20equity). Although formulated primarily for national and international policy, the principle applies to policy relating to all entity sizes, ranging from global policy to family and individual policy, including enterprises of all sizes and services like schools, healthcare itself, etc., and units within enterprises. Bureaucracies must integrate the principle of *Health in All Policies* into their strategic policy making and conduct their activities to minimise any potential harmful health impacts, in particular by causing unnecessary frustration, stress and their collateral effects. Failure to integrate *Health in All Policies* in policy‐making can be discriminatory in nature. All bureaucratic measures should be subjected to a *Health in All Policies* assessment.

### 10.7 Cost Externalisation

Under no circumstances should administrative tasks that can be carried out by administrative entities, with or without minor/modest input, be transferred or delegated to those affected. The introduction of online tools must be accompanied by effective quality control to ensure that they work as intended and do not result in unnecessary effort and frustration for users. All bureaucratic measures should be subjected to a cost externalisation assessment.

### 10.8 Size Proportionality and Expansion of Bureaucracies

The principle of the vital needs of the organisation determines the size and nature of the administration must apply. Any expansion must be fully justified with a convincing cost–benefit analysis for the organisation as a whole.

### 10.9 Privilege, Status, Organisational Symmetry

Members of bureaucracies should not enjoy privileges, such as permanence, not enjoyed by other equivalent members of the organisation; the principle of organisational symmetry must be evident.

### 10.10 Responsiveness, Flexibility and Staffing Skills Related to Needs of the Organisation

Bureaucracies must be responsive to the needs of the organisation and individuals they serve. They must have the necessary competence and technical expertise. To remain up‐to‐date, they need personnel flexibility provided not by growth but by training/developmental opportunities and turnover.

### 10.11 Oversight

An important task of many administrations is oversight and control of budgets, performance, behaviour, etc. But who oversees the overseers? Both groups consist of people, with all the diversity of competence, ego, and behaviour this implies, so it is not to be expected that there will be any difference in contraventions of rules and principles, of aberrant and unacceptable behaviour, between controllers and the controlled. The controllers themselves need oversight. And for their activities to be legitimate, they need to be transparent and accountable, especially to those they control. Those overseeing the controllers need to consist at least in part of those controlled. Controlling is mostly a one‐way system; it needs to become two‐way. This will lead to greater mutual understanding, transparency, accountability and legitimacy in the process of controlling.

### 10.12 Performance Evaluation

Bureaucratic units of organisations should be regularly evaluated (every 3 years is probably optimal): rigorously scrutinised for purpose and efficacy, and judged in the context of optimal performance of the parent enterprise. Best practice must be queried and applied as the benchmark. A key target of this scrutiny must be the identification of unnecessary measures and practices, especially any that may reduce/hinder this performance, including those that may affect workforce morale and motivation (Hinsz 2008). Performance evaluation should be extended to individual members of the bureaucracy and an assessment provided of the personnel needs in relation to the needs of the organisation.

Essential principles of the bureaucracy charter

- No unnecessary or misconceived practices.
- Best for the enterprise.
- Best practice.
- Best for job satisfaction and mental health.
- Stakeholder involvement.
- Duty of care.
- Health in all policies.
- No cost externalisation.
- Symmetrical relationships.
- Transparency and accountability.
- Sustainability.
- Quality control, including software applications introduced.
- Regular evaluation.

## 11 A Mechanism for Ensuring Adoption of and Compliance With the Charter: Central Role of a Bureaucracy Oversight Task Force and Its Remit

A charter is only useful if its principles are implemented and enforced, so a mechanism to ensure implementation and sustained compliance is essential. Bureaucracies are enforcers of regulations, so this element of oversight is nothing new for them.

### 11.1 Creation of a Bureaucracy Oversight Task Force

All organisations should establish a Bureaucracy Oversight Task Force—BOTF—whose task is to drive implementation of the Bureaucracy Charter (see also Frey and Stutzer 2006). Its composition may vary according to organisations and cultures, but should include four types of individuals, namely (i) representatives of the workforce units particularly impacted by bureaucracy, (ii) the leadership who understand well and are responsible for the organisation and its success, (iii) the administration and (iv) the Ombudsperson (see below). Ideally, there should be two members from (i to iii), one of which is elected by the workforce. The BOTF should be chaired by an elected member of the workforce.

### 11.2 Identification of the Most Important Issues to Address

The BOTF should query the workforce at regular intervals on which bureaucratic measures are the most onerous/useless/time‐consuming/inhibitory of productivity/frustrating. This questionnaire might suggest a few obvious examples to initiate the flow of critical juices, but it must specifically seek to stimulate the workforce to articulate all issues of importance. Based on the feedback received from the workforce, and its own considerations of organisation‐specific/cultural aspects that need to be taken into account, the BOTF should establish a priority list of contentious bureaucratic measures, identify improvements that need to be addressed (with concrete examples), and create a road map of actions and issues relating to realisation of the elements of the Bureaucracy Charter (at this point, different experts, e.g., in software solutions, may need to be consulted). This road map should be presented to the organisation leadership for approval of adoption, implementation and progress monitoring by the BOTF.

### 11.3 Involvement of a Trusted Ombudsperson

There will be instances where individuals will want/need to remain anonymous and not (at least initially) be known to the diverse group that will constitute the BOTF and the bureaucracy with which it interacts. Many enterprises have instituted the post of Ombudsperson (OBP), an elected, independent and impartial official whose role is to help people find solutions to problems they experience in the workplace. Organisations currently lacking an OBP should create one. The OBP would receive in confidence complaints and suggestions from individuals and seek solutions either directly with the relevant administration unit or the BOTF. The OBP would be a member of the BOTF.

### 11.4 Monitoring and Reporting

The BOTF is responsible for monitoring the administration and for receiving and dealing with complaints. It should meet regularly to discuss efforts to align goals, optimise procedures and strive for engagement and cooperation. Each meeting should produce a report detailing the issues handled and outcomes. It should regularly articulate its assessment of which procedures (if any) are not essential/of dubious benefit/create irritation/frustration/stress, not best practice‐benchmarked, do not demonstrate duty of care/take into account *Health in All Policies*/observe the requirement to avoid cost externalisation, do not promote a quality culture/team spirit/corporate loyalty. By the same token, progress made and achievement of excellence by the bureaucracy should be recognised and lauded (e.g., https://www.indeed.com/career‐advice/finding‐a‐job/what‐makes‐a‐company‐a‐great‐place‐to‐work).

### 11.5 New Measures and Changes Proposed by Bureaucracies

Any new procedure must, before introduction, be honestly and transparently justified and subjected to an objective and comprehensive cost–benefit analysis, based on rigorous due diligence. This should include how other bureaucracies deal with the issue (comparisons) and which action seems to give the best value to the enterprise/be the least disruptive to its operation (benchmarking). ‘*But what if*?’ is a common justification that frequently has no rational basis and should be discounted unless backed up by compelling evidence. The proposal and cost–benefit results in written form must be submitted to the BOTF and all stakeholders for critical consideration and subsequent collective discussion. The final decision on proposed new changes should reflect the consensus view of this discussion, which should also be documented and the report transmitted to the Performance Assessment evaluators (see below).

Some bureaucratic measures are imposed from outside or at a higher level. These should also be subject to scrutiny by the BOTF. In the case of measures that have questionable justification, or lack compelling justification for their particular enterprise, it is incumbent on bureaucracies—supported by the BOTF and the leadership of the organisation—to vigorously resist their adoption, to push back and, if necessary, demand a rigorous and, above all, relevant cost–benefit analysis from the agency imposing the new measure. Where implementation of a new measure is deemed to be unavoidable, consultation of those affected should nevertheless ensue to assess whether the measure as specified is appropriate for the enterprise, or whether better alternatives exist or can be devised and lodged with the agency imposing the measure as part of the written objection.

### 11.6 Collection and Handling of Personal Data

While organisations have a right to know who they employ and some essential information, personal data collection and management by many organisations exists in the framework/culture of *What if*? i.e., *what if* it might at some point in the future be useful for something or other? The practice of personal data collection must immediately change and be carried out in the context of heightened awareness that there is a reasonable probability that such information will be obtained by hackers, with all of the potential harm this can cause employees. This is an issue of basic duty of care and *Health in All Policies*.

Critical scrutiny of personal data collection and handling should be a specific task of the BOTF, which should assess need by posing the questions app: is the reason for the collection of this piece of information compelling? What would be the consequence for the organisation of not having it?

### 11.7 Reorganisation and Expansion of Bureaucracies

Proposals to reorganise or create new responsibilities/portfolios of bureaucracies should be subject to scrutiny of their justification and cost‐benefit analysis for the organisation by the BOTF prior to implementation. Similarly, expansion of bureaucracies and their resources must be convincingly justified to the BOTF and not be considered in isolation but in relation to personnel needs elsewhere in the enterprise, particularly those of the producing entities (see also Yang and Grenier 2025). The need for an increase in resources, especially personnel resources, must always be considered and judged on the basis of the priorities of the enterprise as a whole. Because of the issue of conflict of interest, particular scrutiny should be directed at justifications of new bureaucratic measures that require, or in future are likely to require, additional personnel. Indeed, Slim (2020), himself a bureaucrat, in relation to existing but “bloated” bureaucracies, wrote: “First, we could apply Ockham's Razor a little more frequently. William of Ockham, a thirteenth‐century Franciscan critic of an overly developed Papal bureaucracy, was a staunch supporter of simplicity in thinking and organisation. His famous rule of Ockham's Razor (McFadden 2021; 2023), insists upon the principle of simplicity and urges that we cut away unnecessary complexity if we can get to the same result with simpler reasoning or resources… In other words, do not hire a new person or build a new team for every new problem or task.”

### 11.8 Annual Performance Reporting

Bureaucracies, like other entities within the enterprise, must provide annual reports on their activities, goals, achievements, failures, challenges, plans for the future, and finances. These must be provided in a form that permits ready comparisons with previous and future performance reports and become a key part of the documentation for performance assessments. The BOTF should discuss and add a written comment‐assessment on it before it is finalised and distributed to the Workforce and the Performance Assessment Evaluators (below). This should be followed by an open Workforce‐bureaucracy‐leadership discussion‐brainstorming to explore potential improvements in bureaucracy functioning.

### 11.9 Regular Performance Assessments

Unlike the enterprises they serve, the performance of bureaucracies is often not reviewed or, if it is, usually by bureaucrats, within a bureaucracy framework, rather than the broader framework of the functioning of the enterprise. This may not provide the level of independence, objectivity and global overview‐vision that is needed. All bureaucracies must be regularly reviewed—every 3 years is probably optimal for most—by a group of external experts and internal stakeholders who should measure past performance and future plans against the needs of the enterprise and health and motivation of its workforce, and assess bureaucracy efficiency, appropriateness of its budget, and its responsiveness to change. Importantly, the perception of their benefit to the different units of the enterprise must be agnostically queried, assessed and documented. *Major goals must be (a) to assess value for the enterprise (productivity, mission success culture‐coherence*, etc.*) of the bureaucratic unit, benchmarked against similar enterprises, identify any activities that provide less value for money than the best, and articulate recommendations for improvement, and (b) to assess workforce wellbeing (*e.g., *periods of illness, absences, satisfaction statistics) and critically compare with data characteristic of the benchmark organisations in the field*. Job descriptions and fulfilment of prescribed tasks of all individuals of a bureaucratic unit must be scrutinised and their validity and necessity for the enterprise they serve critically assessed. The key question that must be posed in this assessment is: *if this function/person did not exist, would the success of the enterprise be materially prejudiced?* Potential conflicts of interest must be identified and scrutinised, for example, in relation to personnel expansion and promotions. In all cases, the assessment should make explicit written recommendations with regard to changes in personnel, budget and responsibilities to be implemented, and for measures undertaken by the bureaucracy. These recommendations, and their subsequent adoption and consequences, should form an integral element of subsequent assessments. Evaluation belongs to fairness and even‐handedness throughout an enterprise.

### 11.10 Another Layer of Bureaucracy?

This proposal obviously introduces further bureaucratic activity into organisations. The key issue is that the explicit mandate of the BOTF is to eliminate unnecessary bureaucracy in an organisation and thereby contribute to a global effort to reduce the diverse costs excessive bureaucracy entails. An appropriate analogy might be that clinical treatment of a sick patient with surgery or medication often adds physical‐metabolic insult to the underlying issue, but with the aim of curing the disease.

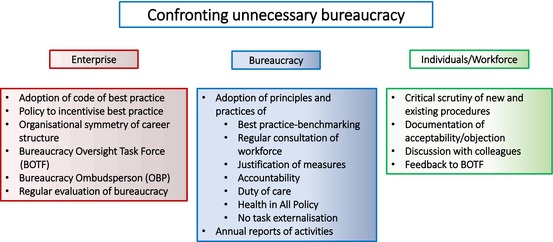

## 12 Organisations Linking Up to Raise Awareness, Synergise, Raise the Bar and Optimise Activities

Different organisations have different cultures and practices, differences that can synergise and enable the evolution of better practices and norms, which in turn raise the bar. BOTFs and OBPs of different organisations might usefully seek to exchange experiences, set standards, develop guidelines and best practices, act as a forum for new ideas, and lobby for adoption of the Charter. Eventually, they might create formal alliances—umbrella organisations—for this purpose. Administrations and bureaucracies should seek involvement in such alliances because they are key stakeholders, have a major contribution to make, and can help shape their future and that of their interactions and impact on the success of their enterprise. Indeed, the best bureaucracies could/should be the pioneers in promoting change among their peers.

## 13 Globalisation of the Bureaucracy Charter: The Utility of an International Body to Galvanise and Coordinate the Actions of National Governments to Address Global Problems

The global scale of unnecessary bureaucracy and its negative impact on international productivity, economics, employee wellbeing and organisational cohesion would undoubtedly benefit from international action to reduce unnecessary bureaucracy globally. This would have the greatest success if it involved world‐class leadership and gravitas that can make abundantly clear the impediment unnecessary bureaucracy represents to the development of solutions to contemporary crises and sustainability, and of advances that improve human and planetary wellbeing, and that can influence, galvanise and channel action by national governments.

## 14 The Blurring of Goal Distinction Between Different Types of Organisation: A Japanese Philosopher's Viewpoint

At the beginning of the 21st century, national universities, hospitals, research institutes, and other public institutions across Japan were converted into independent administrative institutions, a reorganisation that gave each institution the appearance of being entrusted with its own operation and management based on autonomy. However, while this reorganisation allowed universities to be run independently of the government, in reality it left economic management as a corporate entity to the individual institutions. As a result, it became increasingly difficult to pursue freely and creatively activities, such as study and research, education, art and culture, and healthcare, that do not inherently aim to increase profits.

As long as modern nation‐states, as fiscal entities, aim to expand GDP through competition with other countries, rigid and strengthened control of such activities is inevitable and bureaucracies have no choice but to see the successful management of national finances as an end in itself. Scientific research and medicine will inevitably be subject to unnecessary bureaucratic intervention that is based on economic rationality dogma, which creates an excessive increase in administrative work and stress among researchers.

What is needed is to distinguish activities that do not directly aim at profit, such as scientific research, medicine, the arts, and education, from other activities aimed at increasing GDP, and to have the former activities accepted as autonomous entities by states, the United Nations, and other organisations. This will enable such endeavours/enterprises/organisations to develop their own different and independent ecosystems that are fit for purpose and enable them to achieve the specific goals with which they are charged.

## 15 Galvanising the Bureaucracy Ecosystem Reset, and the Predestined Role of Scientists, Academic Institutions and Learned Societies

Perhaps because it is difficult to manage, control, and apply the usual performance metrics to the key attributes of research scientists—creativity, originality, discovery—it seems that managers impose more unnecessary bureaucracy on them than others, though of course this may be an unjustified perception. Nevertheless, the frustration levels about unnecessary bureaucracy in academia are exceptionally high and may constitute significant motivation for improvement of a progressively deteriorating situation. Research scientists, their work, the success of their institutions, and humankind in general may have more to gain through a reduction in unnecessary bureaucracy.

**The special responsibility of academics to catalyse a reduction in unnecessary bureaucracy**

Academics are trained in analytical‐critical‐systems thinking, so are often able to appreciate and understand bottlenecks impeding progress and to develop solution strategies to alleviate them. Most importantly, academics are highly networked, both with academic institution leaderships and other academics—also internationally—and many are networked with peers in industry and government, so this constitutes a favourable framework for galvanising change in academia globally. Academics are also teachers: it is essential to communicate to students the need to evaluate the necessity of bureaucratic measures and not to passively accept without question those that are imposed. Dealing with bureaucracy is part of life and teachers have the obligation to prepare young people for navigation of life's problems. For these reasons, academics have a special responsibility, but also opportunity, to initiate change in bureaucracy and push back against unnecessary bureaucracy.

Most academics belong to learned societies that can inform and coordinate activities of various types, including advising government, public institutions and business. Learned societies often belong to umbrella organisations, such as the International Union of Microbiological Societies (IUMS; https://www.iums.org) and the Federation of European Microbiological Societies (FEMS; https://fems‐microbiology.org). Furthermore, some academics are elected to Academies, such as the European Academy of Microbiology (EAM; https://fems‐microbiology.org/european‐academy‐of‐microbiology/), the American Academy of Microbiology (AAM; https://asm.org/academy/academy), the European Molecular Biology Organisation (EMBO; https://www.embo.org), and the Royal Society (https://royalsociety.org), which are considered to represent some of the best of science, and hence have authority and gravitas. Learned societies, their umbrella organisations and academies have a special duty to lead, to make discoveries and innovate to advance humankind, to solve crises and to warn society and governments about problems, the seriousness of which may not be appreciated, or at least not appropriately reflected in policy (e.g., see https://en.wikipedia.org/wiki/World_Scientists%27_Warning_to_Humanity; https://academic.oup.com/bioscience/article/67/12/1026/4605229; https://www.ucs.org; Cavicchioli et al. 2019; Crowther et al. 2024).

Academics, their learned societies and academies have convening power and must act to begin implementation of the Bureaucracy Charter by

- Engaging the leadership of their institutions.
- Identifying the impediments of unnecessary bureaucracy and explaining their consequences.
- Galvanising the creation and activities of BOTFs.
- Activating their networks, nationally and internationally. Volunteering as members of BOTFs, umbrella organisations, and international organisations for the elimination of unnecessary bureaucracy.

*The time has come for academics to transition from complaint to solution and play their role in planting unnecessary bureaucracy on the global agenda!*

## 16 Development of a Better Culture‐Ecosystem

Creating a better workplace ecosystem‐culture and better mutual understanding will undoubtedly have substantive benefits including the all‐important team spirit, associated motivation (Ryan and Deci 2000; Hinsz 2008) and sense of collective achievements (see also Landry 2020). Below we list some ways of achieving this:

### 16.1 Supporting and Valuing Good Bureaucracy That Strives for Best Practice

Frustration and resentment are not one‐sided: some members of bureaucracies may also feel unappreciated and undervalued, and others may not perceive career advancement possibilities. These perceptions may contribute to (though not excuse) some of the problems and practices discussed above. It is vital that good work everywhere, including that of bureaucracies, be appropriately appreciated and rewarded, including through a timely “thank you”. Greater effort must be made by all to understand the problems and frustrations of bureaucracies, and to value good work whenever it is evident. The development of transparent, merit/performance‐based career advancement routes is essential.

### 16.2 Placing the Wellbeing of Others at the Centre of Bureaucratic Policy and Practices

The principles and practices of *duty of care* for individuals within and affected by bureaucracies, *Health in all Policies*, and of prohibiting the practice of *externalisation of costs*, especially non‐economic costs, must become embedded in the psyche, functioning and standard operating procedures of bureaucracies.

### 16.3 Creating Awareness and Openness, and Rehabilitation of the “Why” Question

One of the routes to better relationships between bureaucracies and frontline workers is improving openness and dialogue about issues that grate and potential measures to reduce or eliminate them. Creating a forum for discussions of both acute and chronic issues on both sides can facilitate better mutual understanding and the search for solution strategies. A key element of this is encouragement‐incentivisation of a culture of questioning—why?—and exploration of alternative, sometimes creative measures.

### 16.4 Replacing a “Them and Us” Attitude With a Stakeholder Culture of Shared Responsibility

Some problems between bureaucrats and frontline deliverers are exacerbated by a culture of “them and us” which obviously has no place in an organisation seeking success and achievement and in which working together for maximum productivity is essential. Much greater effort is needed to involve all stakeholders in policy development and implementation, to develop a sense of shared responsibility, and to escape from blame culture (Landry 2020).

### 16.5 Making Everyone Accountable

Shared responsibility also means acceptance of blame for mistakes. For this to be fair, transparency and recognition of the need for accountability are essential.

### 16.6 Creating Incentives for Improved Procedures, Benchmarking, and Adoption of Best Practice

A source of resentment is that some bureaucracies develop their own solutions and measures to generic problems for which more effective solutions may already be known. This is “silo thinking” and failure to benchmark. Best practice and benchmarking (https://paulspector.com/bureaucracy‐can‐be‐stressful/) must be central principles of organisations, including their bureaucracies, and should be incentivised.

### 16.7 Improving Understanding, Increasing Social Contact in the Workplace, Development of Friendships

One of the most effective ways of improving understanding between people is to promote social interactions and exchanges, so efforts should be made to create opportunities in the workplace for social interactions, some of which may lead to social interactions outside of the workplace and even friendships.

## 17 Importance of Education and Public Debate

Bureaucracy is central to society and affects everyone. Being able to distinguish essential and non‐essential bureaucracy is vital, not only for understanding the role and importance of bureaucracy in the safe and effective functioning of society, in the same way that healthcare, education, food security, justice, etc., play such a role, but also for constructive discussions and actions to deal with unnecessary bureaucracy. It is also crucial that pervasive excessive bureaucracy does not become the norm, and hence accepted, which is a real danger for the next generations that grow up in over‐bureaucratised workplaces and societies. The role of bureaucracies in society must be taught in school and historical examples of excessive bureaucracy at different levels and their societal consequences explored and critically discussed. This should be accompanied by a wider debate in society, the body politic, International and Non‐Governmental Organisations, commerce and healthcare about which kinds of bureaucratic ecosystems society needs.

## 18 Role of Global Bureaucracy in Confronting Global Challenges

### 18.1 Global Challenges Facing Humankind

Humankind currently faces an unprecedented number and spectrum of challenges, some of which are existential, issues that urgently need solutions/mitigating strategies. These crises may be local, regional or global and include global warming predicted to cause 14.5 million premature deaths by 2050 and 2 billion disability‐adjusted life years (DALYs), with children and the elderly in low‐resource settings being disproportionately affected (IPCC 2023; WHO 2023; Bishen and Glick 2024), pollution (young children are particularly vulnerable to air pollution ‐ it is estimated that around 2 billion live in areas burdened with air pollution levels that exceed WHO limits https://www.unicef.org/sites/default/files/2019‐02/Clear_the_Air_for_Children_Executive_summary_ENG.pdf), antimicrobial resistance (AMR, which is predicted to cause the loss of 10 million lives per annum by 2050; O'Neill 2016), poverty, food insecurity, etc. Satisfying the needs of an exponentially increasing human population (Cohen 1995) involves a growing rate of extraction‐utilisation of natural resources – “the great acceleration” (Steffen et al. 2015), and reallocation of planetary resources, creating potentially catastrophic imbalances and resulting loss of biodiversity and ecosystem integrity and functioning (Baquero et al. 2026). Global human‐made mass, principally construction materials used in buildings and infrastructure, now exceeds all living biomass (Elhacham et al. 2020; Greenspoon et al. 2025).

### 18.2 The Asymmetry in Problem Growth and Solution Finding, and the Key Role of Discovery and Innovation

While policies such as the Sustainable Development Goals of the United Nations continue to be developed by various players, their implementation and the associated societal behavioural changes they necessitate are taking place too slowly and too late (United Nations 2022, 2025). Whereas some problems and crises can be addressed by available solution strategies, if supported by appropriate political decisions and the necessary investment, others require new solutions that depend upon advancing knowledge which itself is dependent upon original and creative discoveries made by researchers. For example, there are a number of promising innovative approaches to counter AMR but progress is slow and mortalities from infections caused by AMR pathogens are rising. The same is true of advances in agriculture that will reduce starvation and improve human nutrition, pollution mitigation, etc., that will similarly reduce avoidable morbidities and premature mortalities. Avoidable morbidities, premature mortalities, avoidable hunger, and avoidable exposure to pollutants constitute avoidable human suffering (Timmis et al. 2025) which is also associated with significant collateral implications, such as family fragmentation and stress with consequences for healthcare, within family and inter‐family care, children's responsibilities and education, stress and mental health, and so forth, in addition to impacts on the demographic dividends and national and global economies.

### 18.3 Solution Finding Is Directly Impacted by Bureaucracy

Effectively confronting current challenges requires a sharp focus on underlying causes and potential solutions with maximum efficacy and productivity, and that respect the requirements of sustainability, a step increase in efforts of everyone to deal with them, and targeted elimination of unnecessary hindrances at all levels that slow progress. A marked acceleration in discovery, innovation and implementation, creation of more favourable political, regulatory and legal frameworks, and education and persuasion of society about necessary changes, is essential for policies to achieve their goals. The activities of organisations that contribute to the discovery and implementation of new or better solution‐mitigation strategies and measures are directly and indirectly impacted at different levels and to different degrees by varied and multiple bureaucratic controls. Bureaucracy plays a nodal role in the efficacy and efficiency of human endeavours and has a significant role in determining the speed of progress made by key players (see example of permitting processes hampering Europe's energy transition: Ferris 2022; Affre and Olivieri 2024; Piotrowski and Gislen 2024). Supportive bureaucracy promotes solution finding, excessive bureaucracy constitutes a significant impediment.

### 18.4 Bureaucracy and the Efficacy of National and International Organisations That Implement Solution Strategies for Global Problems‐Opportunities‐Sustainability

Delivering solutions to critical challenges will generally involve the participation of multiple organisations, international and national, Non‐Governmental Organisations, and smaller enterprises and research operations, all of which have bureaucracies. Those organisations with excessive bureaucracies will not be able to realise their full potential in solving global, or indeed regional or community problems (Vaubel 2006; Butler 2013; Slim 2020). International organisations need citizens' oversight and control (Frey and Stutzer 2006).

## 19 Concluding Remarks

We freely, gladly and gratefully acknowledge and appreciate that many bureaucracies and administrations fulfil some or most of the principles of the Charter articulated above. There are many dedicated individuals who carry out vital duties that ensure the success of their enterprise in almost all bureaucracies and administrations (see also Landry 2020). They are the benchmark and we salute them! This Editorial is not therefore directed at such bureaucracies and their members, except in the sense that their support and involvement in efforts to raise standards will accelerate vital change. They certainly have a vested interest in an improvement in the image of bureaucracies in general.

The purpose of this Editorial is to induce bureaucracies and bureaucrats, wherever they are and whatever their organisation does, to improve practices, attitudes and work ethics through benchmarking and regular consultation of stakeholders. It is not to demonise, nor add to existing demonisation: quite the contrary, our purpose is to promote the creation of workplace ecosystems characterised by better integration and cohesion of the different activities of organisations, better understanding of the roles, activities and problems of different organisational units, and improved mutual respect between interacting individuals and groups in the workplace. It is to promote team spirit, individual wellbeing, and optimal productivity (https://www.indeed.com/career‐advice/finding‐a‐job/what‐makes‐a‐company‐a‐great‐place‐to‐work). The elimination of unnecessary bureaucratic measures will go a long way towards achievement of this goal. To raise awareness of the issues at stake, we have formulated a Bureaucracy Charter and a mechanism to ensure its implementation as intended. The trio of Charter, Bureaucracy Oversight Task Force, and Ombudsperson will bring clarity and focus to obligations and provide a format for constructive dialogue.

Our purpose is also to galvanise action by drawing attention to the global magnitude of the problem of unnecessary bureaucracy and through exploration of some of the potential global consequences. It has been reported that there are approximately 360 million businesses worldwide, plus around 10 million non‐profit organisations, plus governmental and intergovernmental organisations (https://www.worldbank.org/en/programs/entrepreneurship/total‐number‐of‐firms; https://www.demandsage.com/business‐statistics/; https://techreport.ngo/previous/2017/facts‐and‐stats‐about‐ngos‐worldwide.html). While many of these organisations are small with little or no bureaucracy, and others will have lean bureaucracies that impose little or no excessive measures, there are millions of organisations with bureaucracies that impose unnecessary measures that reduce organisational productivity. The global detrimental impact of excess bureaucracy on the global economy, humankind's wellbeing and efforts to solve and mitigate global and regional challenges is likely to be immense. Moreover, it is essential to appreciate the growing, aggregate impact of excessive bureaucracy—its epidemic nature—as a growing crisis. That this is not obvious is because bureaucracy is broadly accepted as an essential positive force, and problems of unnecessary bureaucracy tend to be experienced by individuals and groups of individuals, so often remain personal and local. The global importance of excessive bureaucracy is largely unappreciated and not on the radar screens of most world leaders. However, addressing global unnecessary bureaucracy will undoubtedly yield significant global benefits. It is therefore essential to make apparent the scale of the problem and its consequences in order to galvanise global action to improve the situation.

A first consideration is productivity: unnecessary bureaucracy diverts time and effort away from primary tasks, reducing productivity. In business, reduced productivity is associated with lower competitiveness and its impact on commercial success. In government, reduced productivity may be reflected *inter alia* in reduced societal services; in international organisations and non‐governmental organisations, it may lower achievements in improving humanitarian conditions; in healthcare, reduced disease prevention and healing; in research and development, lower creativity, discovery rates and innovations that improve the human condition or solve problems, etc. A second consideration is the impact of frustration and dissatisfaction on stress creation, and impacts of stress creation on health, interpersonal interactions and support networks. A third consideration is the impact on global efforts to reduce poverty and societal inequalities, global healthcare, and potential consequences for sustainable development. In this regard, it is important to keep in mind that the various strands of consequence are intertwined. For example, unnecessary bureaucracy‐created stress‐induced health disorders, reduced productivity in healthcare systems, and reduced healthcare‐relevant discoveries and their translation into clinical practice through reduced creativity and innovation in research and development will all impact global healthcare, human suffering, life spans and associated societal inequalities. Similarly, different strands impact sustainable development (e.g., https://news.mongabay.com/2013/01/bureaucratic‐reform‐plays‐a‐part‐in‐reducing‐deforestation‐in‐indonesia/). In order to obtain a comprehensive overview of the global consequences of unnecessary bureaucracy, research dedicated to quantitative assessment of these potential impacts is urgently needed.

**Research needs**

World leaders need reliable information on parameters that influence productivity, innovation, workplace health and sustainability. Collectively, bureaucracies have major and cumulative impacts on global issues, impacts that need to be delineated and effectively addressed. Research is urgently needed on the following aspects, and others, of the impacts of unnecessary bureaucracy on:

1. The productivity of organisations.
2. Stress and stress‐induced health issues in organisations.
3. Global productivity, the global economy (annual; cumulative).
4. Resource consumption/wastage, sustainability (annual; cumulative).
5. Global health and healthcare costs (annual; cumulative).
6. Global efforts to reduce inequalities.
7. Creativity‐discovery‐innovation‐implementation of technical solutions to global problems.
8. The capacity to optimally train a future workforce that is innovative, curious, creative, analytical, critical, rigorous and professional.
9. Gut microbiome compositions and functionalities, and their relationships to stress, ill health and behaviour.

It is to be expected that the solution strategy proposed here will meet some resistance. This is natural because change almost always provokes resistance. But change can be achieved if the reasons are compelling and convincingly explained. The research needs listed above will undoubtedly provide valuable additional evidence to complement that which is currently available. However, mobilisation of a grassroots movement is needed. Everyone who feels the need to reduce unnecessary bureaucracy should engage, discuss with their fellows, identify and bolster their case with clear examples of unreasonable bureaucratic measures that hinder their productivity, and seek contact with their bureaucracy and leadership. They should also seek contact with like‐minded groups in other enterprises to join forces, share experiences and increase the pressure for change.

There is another important aspect that will facilitate acceptance of the solution strategy proposed here. Unlike other global crises, such as global warming, the fixing of which may require politically challenging outlays of taxpayer revenues and behavioural changes in large segments of the population, solving the unnecessary bureaucracy crisis will not entrain significant costs. The explicit purpose of the Charter and its implementation mechanism is to increase productivity and save costs, so it is unlikely to experience significant push‐back from society—quite the contrary—and will actually save taxpayer revenues. Developing policies to constrain excessive bureaucracy should increase political advantage rather than constitute a handicap.

It is time to put unnecessary bureaucracy on the global agenda. It is essential to change culture from one of passive receivers of instructions to team members involved in decisions; this will *inter alia* empower future generations and their ability to decrease inequalities.

## Author Contributions

**Kenneth Timmis:** conceptualization, writing – original draft, writing – review and editing. **Zeynep Ceren Karahan:** writing – review and editing. **Jennifer A. Byrne:** writing – review and editing. **Jake M. Robinson:** writing – review and editing. **Patricia Bernal:** writing – review and editing. **Paul B. Rainey:** writing – review and editing. **Purificación López‐García:** writing – review and editing. **Terry J. McGenity:** writing – review and editing. **Max Chavarria:** writing – review and editing. **Kazuo Sato:** writing – review and editing. **Willy Verstraete:** writing – review and editing. **Lars M. Blank:** writing – review and editing. **Rup Lal:** writing – review and editing. **María Francisca Colom:** writing – review and editing. **Juan Luis Ramos:** writing – review and editing.

## Funding

The authors have nothing to report.

## Conflicts of Interest

The authors declare no conflicts of interest.

## Data Availability

This is an Editorial and contains no newly generated data.

## Appendix 1 Examples From Academia of Unnecessary Bureaucracy

Example 1: *Unnecessary financial burden*—*Reimbursement of travel expenses*
Learning about current developments in other laboratories working on related projects and, equally importantly, unrelated topics, brainstorming with colleagues and building relationships that lead to reciprocal exchange of newest tools and materials, and promoting own work, is vital for rapid progress and hence effective use of taxpayer funding of research. While remote attendance of conferences is possible and increasing, it does not allow the same degree of spontaneous interaction that leads to exploration of new ideas, so regular in‐person attendance and face‐to‐face discussions are essential.
*Problem: Unreasonable financial burden on early career scientists*. In many institutions, attendance of conferences involves attendee payment of registration fees, hotel accommodation, meals and travel costs, which are subsequently reimbursed following submission of a reimbursement application. The time taken for reimbursement usually takes weeks, sometimes months. Registration costs often increase closer to the conference, so early registration, often 4–6 months before the event, is required to obtain the best rate. Air fares also increase closer to the day of departure, so they need to be booked well in advance. Conference organisers usually make block bookings of hotel rooms at reduced rates but participants need to make reservations well ahead of the conference in order to secure a room. All of these actions save substantial taxpayer money, but entrain significant financial outlays by the attendee and long periods of time – 3–6 months is not unusual – before the outlays are reimbursed. Outlays approaching €/$/£ 2000 are not unusual (registration‐hotel‐airfare: 600‐600‐600). While senior scientists may be able to carry this burden without too much problem, early career scientists who may have net incomes of €/$/£ 2000 per month may experience significant financial difficulty, *in effect a lack of one month*'*s income for a period of several months*. The bureaucracy of reimbursement of financial outlays has as its purpose correct financial management and keeping costs as low as possible. But it often does this by placing an enormous financial burden on the attendee. In our view, this financial burden is unnecessary.
*Solution: the admin could pay such costs directly, or reimburse outlays instantly on the understanding that the reimbursement is only provisional and subject to a proper accounting after the meeting. Indeed, this is practised in some institutions, so it is entirely practicable*.
*Problem: Excessive bureaucratic burden for small sums*. In extreme situations, people do not go to conferences or stop applying for funding because of the workload accompanying these activities. On one hand, this is improper because they have an obligation to publicly share new results; but, on the other hand, this is associated with significant bureaucracy‐caused loss of research time and considerable frustration.
*Solution*: a sliding scale of bureaucratic oversight proportional to the sum involved in the transaction, i.e., a simple generic form for all transactions, with additional forms requiring further information for transactions involving larger sums.

Example 2: Excessive form filling In a recent EC‐funded grant to a national government, it is necessary to fill out 9 documents to request a reagent – one of the documents is a declaration of no conflict of interest with the company to which the request is made.
*Problem*: While this level of bureaucracy may be justified for transactions involving large sums of money, for transactions involving small sums, sometimes less than €10, there is no relationship between the level of bureaucracy and its purpose, with the consequence that the effort involved by both the researcher and the administration is time wasted, with corresponding loss of productivity, and hence wastage of tax resources.
*Solution*: a sliding scale of bureaucratic oversight proportional to the sum involved in the transaction, i.e., a simple generic form for all transactions, with additional forms requiring further information for transactions involving larger sums.

Example 3: Irrational grant management
*Problems*:With regard to one funding agency, we must prepare grant applications in English because there are too few national reviewers, so foreign reviewers are sought. If the application is successful, it has to be submitted again in the national language. It is not read by the grant agency (apart from the budget) but the language of the agency is the national language. The period between application submission and project start is 1 to 2 years. Nevertheless, it is necessary to specify which conference in which year will be attended and the transport and hotel costs associated with it, even though these are not usually known at the time of grant submission. If it becomes necessary to attend another conference later on, it is necessary to make a new application and the existing travel funds cannot be used for it. Depending on the grant agency, consumable materials needed must be specified in detail, as well as how and when they will be used. Once the budget is approved, there is little flexibility to change elements in it in response to unanticipated needs which always arise in research. The university as an interface has different administrative requirements than the funder, and spending criteria of the funding agency are so tightly knit that they cannot be implemented by the university. As a result, some funds may have to be returned even though they could have been put to good use. Approval of funding is notified at such short notice that it is impossible to hire staff for the start of the project. However, the funding period is fixed so that funds may be forfeited and, for example, completion of a doctoral project may have to be financed from other sources.
*Solution:* common sense and alignment‐integration of procedures between funding agencies and the official recipients who manage the awarded funding.

Example 4: Disproportionate time and effort needed to gain regulatory approval
*Problem*: “For a 1‐year, negligible‐risk project examining biobank operations conducted at three separate national institutions, we found that the researcher time required to meet regulatory requirements was eight times greater than that required for the approved research activity (60 h versus 7.5 h, respectively; Rush et al. 2018)”. The consequences of this are that some tasks are not done and the cost of others becomes more expensive.
*Solution*: Common sense based on proportionality.

Example 5: Dysfunctional online tools
*Problem*: The ubiquitous development of online tools is supposed to alleviate bureaucratic burden but mostly has only increased it and amplified frustration. These tools are often specific to different types of institutions and funding bodies, so it is necessary to take the time to learn how to navigate different systems that basically have the same purpose. Moreover, they are rarely user‐friendly, and many informatic bugs are the norm. When things become blocked, the system doesn't tell you what is wrong, so you don't know where to look. Often, there are multiple checkpoints involving 2–4 persons (secretary, team leader, administrator, unit director) who need to validate with a click somewhere. In one funding system, you need three different programmes to get your travel organised: one to create the “mission”, another to organise the expenses for the travel, and yet another one to buy your tickets or make your hotel reservation. Now that everything is online, you lose much more time and energy than before and, when problems arise, it is very difficult to know what they are and how they can be solved. You end up annoying many people until you discover, adding stress to everyone.
*Solution*: Proper quality control.

Example 6: Nonsensical Procedures Rooted in Absolutism Instead of Proportionality
*Problem*: To send a conventional letter by post, it was first necessary to fill out a form and send it to the university administration for approval of the expense. Approval was subsequently communicated by inter‐campus mail. The cost in materials and staff time (and the unnecessary delay incurred) expended to manage the trivial expense of letter postage was significantly more than the amount being controlled.
*Solution*: Fortunately, common sense, but above all the significant decrease in the number of letters sent by post, led the institution to eliminate the measure.

Example 7: Arbitrary Deadlines Uncoupled From the Purpose of the Task They Regulate At many universities and research centres, researchers are required to document their research achievements at regular intervals, typically every 5 years. Previously, researchers complied by completion of a form that was sent directly to a national evaluation commission at the beginning of the year following the evaluation period.
*Problem*: This procedure changed recently and submissions must now be made through the university library. The deadline for deposition of evaluation dossiers set by the administration is now before the end of the evaluation period, which prevents inclusion of articles published towards the evaluation period. This is particularly frustrating because humans typically work to deadlines and the end of the evaluation period is the most active time for publication. While there are always unanticipated delays which can push publications into the following year, the arbitrary cut‐off of the last part of the year can seriously prejudice performance documentation. Bureaucracy takes precedence over the purpose of the procedure, which is the evaluation of research activity.
*Solution*: The deadline for inclusion of evaluation materials must be the end of the evaluation period.
